# *PsGA2ox2* is a novel target of miR159 involved in endodormancy regulation in tree peony (*Paeonia suffruticosa*)

**DOI:** 10.1186/s43897-025-00220-9

**Published:** 2026-05-12

**Authors:** Wenyu Gai, Xinyu Wang, Yongli Zhao, Tao Zhang, Yanchao Yuan, Chunying Liu, Shupeng Gai, Yuxi Zhang

**Affiliations:** 1 College of Life Sciences, Qingdao Agricultural University, Qingdao, 266109 China; 2 University Key Laboratory of Plant Biotechnology in Shandong Province, Qingdao, 266109 China; 3 College of Horticulture, Qingdao Agricultural University, Qingdao, 266109 China; 4 Department of Ornamental Horticulture, College of Horticulture, China Agricultural University, Beijing, 100193 China; 5 Shandong Provincial Key Laboratory of Microbial Resource Exploration and Innovative Utilization, Qingdao, 266109 China

**Keywords:** Tree peony, Endodormancy, Gibberellic acid, PsGA2ox, PsmiR159b

## Abstract

In deciduous woody plants, buds enter endodormancy in late autumn, and endodormancy release (EDR) is a essential for regrowth. In tree peony, endodormancy is a major obstacle to forcing culture, an important component of its industry. Both chilling accumulation and gibberellin acid (GA) application can contribute to break dormancy and facilitate bud sprouting and flowering under appropriate conditions. PsmiR159b is one of the most abundant miRNAs in dormant buds, targeting *PsMYB65* to inhibit bud EDR. Here, *PsGA2ox2* was identified as a novel target of PsmiR159b according to expression data and genetic analysis. *PsGA2ox2* expression was up-regulated by prolonged chilling, following with an increase of gibberellic acid 34 (GA_34_), a product of deactivated GA_4_ by GA2-oxidase. Silencing of *PsGA2ox2* accelerated budbreak, accompanied by the increase of GA_1_ and GA_4_, downregulation of *PsABI5*, *PsSVP*, and upregulation of *PsEBB1*, *PsEBB3*, and *PsCYCD*. The content of GA_4_ was downregulated in STTM159b buds, whereas increased in MIR159b-OE buds. In addition, the ectopic expression of *PsGA2ox2* in Arabidopsis dramatically delayed seed germination and inhibited plant growth. These findings indicated a dual-target regulatory role for PsmiR159b in regulating GA homeostasis, and also revealed a PsmiR159b-*PsGA2ox2* module that influenced EDR in tree peony through bioactive GA level regulation.

**Supplementary Information:**

The online version contains supplementary material available at 10.1186/s43897-025-00220-9.

## Core

*PsGA2ox2* is a novel target of PsmiR159b, which is induced by chilling accumulation and maintains bud dormancy by reducing bioactive GA levels in tree peony. PsmiR159b is a novel bidirectional regulatory factor to regulate GA homeostasis during dormancy release.

## Gene & accession numbers

Gene sequence data were acquired from the tree peony genome database (BioProject ID of PRJNA720276 and Genbank accession number of CNP0003098) and The Arabidopsis Information Resource (https://www.arabidopsis.org/). The accession numbers of the genes used in this study are as follows: AtGA2ox1 (AT1G78440), AtGA2ox2 (AT1G30040), AtGA2ox3 (AT2G34555), AtGA2ox4 (AT1G47990), AtGA2ox5 (AT3G17203), AtGA2ox6 (AT1G02400), AtGA2ox7 (AT1G50960), AtGA2ox8 (AT4G21200), AtGA2ox9 (AT5G58660), AtGA2ox10 (AT3G47190), PsGA2ox2 (MH546119.1), PsGA2ox8.1 (JI456918), and PsMYB65 (MT211968).

## Introduction

Some temperate woody species enter endodormancy in autumn, induced by short days (SD) and/or low temperatures, to avoid damage from winter stress. Bud dormancy goes through four stages: endodormancy establishment, maintenance, release, and budbreak (Yang et al. 2021). During endodormancy maintenance, callose accumulation blocks plasmodesmata, limiting the transport of nutrients, water, and signaling molecules, and suppressing metabolic and physiological activities in woody plant buds (Savage and Chuine 2021). The transition from dormancy maintenance to release needs sufficient chilling accumulation (Singh et al. 2016). Recent studies have demonstrated that bud endodormancy release (EDR) is governed by an integrated network involving material transport, plant hormone signaling pathways, genetic and epigenetic regulation, and carbohydrate metabolism (Zhang et al. 2020; Yang et al. 2021; Chen et al. 2022; Xu et al. 2022; Pan et al. 2023).

Plant hormones play a vital role in EDR (Wen and Zhong 2016; Ali and Baloch 2020), especially the antagonism and balance between gibberellin acid (GA) and abscisic acid (ABA) (Tylewicz et al. 2018). Exogenous GA application can partially substitute for chilling to promote EDR (Zhang et al. 2021). Endogenous ABA levels rise during dormancy onset but decline during chilling-induced EDR, while bioactive GA levels increase concomitantly (Zhang et al. 2020). Several ABA-responsive transcription factors, including ABA INSENSITIVE 5 (ABI5) and ABA RESPONSIVE ELEMENT-BINDING FACTORs (ABFs), have been identified as dormancy regulators (Yang et al. 2020). In hybrid poplar, extended cold exposure reduces ABA levels and *SHORT VEGETATIVE PHASE -like* (*SVL*) expression and upregulates GA biosynthesis and *FLOWERING LOCUS T1* (*FT1*) expression, ultimately promoting bud EDR (Singh et al. 2018). In poplar, *EARLY BUD-BREAK 1* (*EBB1*) is activated by chilling accumulation, which directly inhibits the expression of *SVL*, promotes *Cyclin D3* (*CYCD3*) expression, and accelerates budbreak with the assistance of EBB3 (Azeez et al. 2021). Simultaneously, Glucan Hydrolase family 17 (*GH17s*), also known as *1,3-β-Glucanase* genes (*BG*s), are induced by GA and chilling, reopening signal transduction pathways in the embryonic shoot, and facilitating EDR in poplar or tree peony (Rinne et al. 2011; Gao et al. 2021a).

In plants, bioactive GAs are involved in numerous physiological processes, including seed germination, stem elongation, and leaf extension (Macmillan 2001), primarily by promoting cell division (Emamverdian et al. 2020). GA homeostasis is principally maintained by strict regulation of GA-activating enzymes including GA20-oxidase (GA20ox) and GA3-oxidase (GA3ox), and GA-deactivating enzyme like GA2-oxidase (GA2ox).

GA2ox belongs to the 2-oxoglutarate-dependent Fe (II) oxygenase (2OG-Fe (II) oxygenase) subfamily and is defined by a conserved 2OG-Fell_Oxy domain (Zhu et al. 2023), which convert GA_1_ and GA_4_ to their inactive catabolites GA_8_ and GA_34_, respectively (Sun 2011). GA2oxs are involved in seed germination, plant height, dormancy release, and flower development by participating in GA metabolism and signaling (Hedden and Thomas 2012; Liu et al. 2022a, b). In Japanese apricot, *GA2ox* expression is upregulated during dormancy (Yamane et al. 2008). Overexpression of *AtGA2ox7*, *AtGA2ox8*, and *AtGA2ox1* decreases endogenous active GA levels and delays flowering (Schomburg et al. 2003). Similarly, *PtGA2ox* overexpression leads to dwarfing traits and early flowering in poplar (Zawaski et al. 2011). In sweet cherry, PavGA2ox-2L interacts with PavDWARF, a GA negative regulator, to maintain dormancy, suppress plant height, and delay flowering (Liu et al. 2022a, b). Considering EDR is a prerequisite for flowering in deciduous woody plants, these findings suggest a potential role for GA2ox in regulating EDR. However, the regulatory mechanism of *GA2ox* in maintaining dormancy remains unclear.

MicroRNAs (miRNAs) are 20–22 nucleotides that typically regulate gene expression in plants by sequence-specific cleavage of target mRNAs (Loreti and Perata 2022). MiRNAs are involved in many biological processes, including responses to abiotic stress and plant development, such as leaf and flower induction (Khraiwesh et al. 2012; D'Ario et al. 2017). In Arabidopsis, miR165/166 targets the *HD-ZIP* III transcription factor family, which directly actives the expression of *GA2ox* to regulate plant height (Zhao et al. 2022). The miR159 family is the most conserved and abundant across plant species, primarily targeting *GAMYB-like* to regulate physiological processes, such as vegetative growth, male reproductive development, anther, fruit and seed development, flowering, and both abiotic and biotic stress tolerance responses (Millar et al. 2019).

Recently, miRNAs are identified to play a role in the regulation of bud endodormancy (Zhang et al. 2018, 2023; Andrés 2021). In pear (*Pyrus pyrifolia* ‘Kosui’), miR6390 promotes EDR by cleaving *Dormancy-associated MADS-box* genes (*DAM*) (Niu et al. 2016). MiR169 interacts with *PmRGL2* to initiate dormancy release in Japanese apricot by regulating NUCLEAR FACTOR-Y subunit A (NF-YA) (Gao et al. 2021b). In tree peony, miR172 targets to *TARGET OF EAT 3* (*TOE3*) and inhibits EDR by repressing *EBB1* expression (Zhang et al. 2023). miR159 and its targets may be involved in bud dormancy regulation of apple (Garighan et al. 2021) and *Pyrus pyrifolia* (Liu et al. 2022a, b). However, it remains unclear whether any miRNA directly regulates GA levels through targeting GA metabolism-related genes.

Tree peony (*Paeonia suffruticosa* Andr.) is a culturally and economically important ornamental plant native to China. It undergoes endodormancy in winter, which can be broken only after adequate chilling accumulation (Huang et al. 2008; Gai et al. 2012). However, the increasing incidence of warm winters has led to insufficient dormancy release, resulting in reduced sprouting rates and flowering quality. Therefore, understanding the mechanism of EDR in tree peony has become crucial. Our studies have revealed that EDR is accompanied by activation of the Embden-Meyerhof-Parnas (EMP) pathway (Zhang et al. 2020), reopening transport corridor (Gao et al. 2021a), a reduction in DNA methylation (Xin et al. 2019), and activation of GA signaling (Gai et al. 2013; Zhang et al. 2021). Notably, PsRGL1 (RGA-LIKE 1), a key DELLA protein, is degraded after polyubiquitination, resulting in EDR (Gao et al. 2023). Recently, we have demonstrated that prolonged chilling downregulates PsmiR159b in tree peony, and PsMYB65 serving as its target activates *PsCYCD3;1* expression to resume bud growth (Zhang et al. 2024). Based on the expression patterns of PsmiR159b, we hypothesized that it may target additional, yet unidentified transcripts to coordinate EDR.

To verified this, PsmiR159b was used to blast dormant bud transcriptome of tree peony (Zhang et al. 2021), and *PsGA2ox2* as a novel target was obtained. During chilling-induced dormancy release, the transcript levels of *PsGA2ox2* increased to maintain bud dormancy by reducing bioactive GA_4_ levels, correlated with the expression of PsmiR159b. These finding suggested that PsmiR159b potentially functioned as a dual-function regulator of GA homeostasis on dormancy regulation in tree peony.

## Results

### Expression patterns of PsmiR159b and *PsGA2ox*

In our recent study, we have demonstrated that PsmiR159b-PsMYB65-*PsCYCD* module participates in budburst in tree peony. Interestingly, the expression of PsmiR159b is relatively low in non-chilled buds (Zhang et al. 2024), which can hardly be explained by the this module alone. This result prompted us to explore additional potential targets of PsmiR159b during dormancy. Using the mature sequences of PsmiR159 to query the dormant bud transcriptome in tree peony (Zhang et al. 2021), and a *GA2ox2* homologue was hit.

**Fig. 1:**
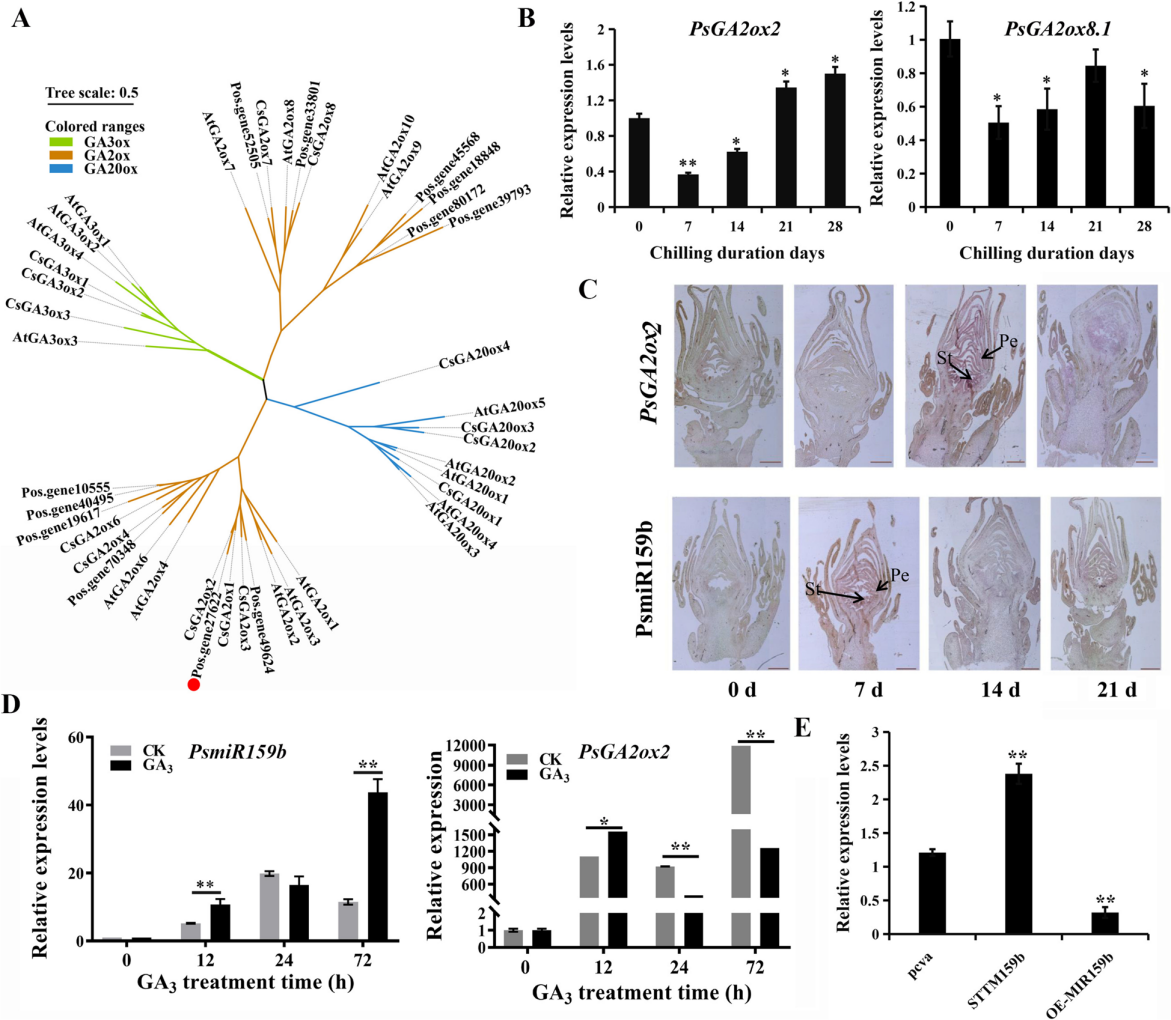
The phylogenetic tree and expression analysis of PsmiR159b and *PsGA2oxs*. **A** The phylogenetic tree of PsGA2oxs and the other plant GA2oxs. At, *Arabidopsis thaliana*. Cs, *Camellia sinensis*. PsGA2ox2 was marked with a red dot. **B** The expression patterns of *PsGA2ox2* and *PsGA2ox8.1* during chilling-induced bud dormancy release using qPCR. Error bars indicated standard deviation (SE, *n* = 3). Asterisk indicated the significant differences (Student’s *t*-test, *P < 0.05, **P < 0.01). **C** RNA in situ hybridization of PsmiR159b and *PsGA2ox2* in buds after chilling treatments. St, stamen; Pe, petal. **D** The expression patterns of PsmiR159b and *PsGA2ox2* after GA_3_ feedings using qPCR. Error bars indicated SE (*n *= 3). Asterisks indicated statistically significant differences (Student’s *t*-test, *P < 0.05, **P < 0.01). **E** Relative expression levels of *PsGA2ox2* in OE-MIR159b and STTM159b buds. pCVA, empty vector control; OE, overexpression; STTM159b, silencing of PsmiR159b. Values were represented as the mean ± SD of three biological replicates. Asterisk indicated the significant differences (Student’s *t*-test, **P < 0.01)

To characterize GA2ox homologues in tree peony comprehensively, AtGA2oxs from TAIR were employed as query sequences in a local BLAST search against the tree peony genome (Yuan et al. 2022), and totally 12 putative PsGA2ox genes were obtained. They clustered into one group homologous to AtGA2ox1, AtGA2ox2, and AtGA2ox4, three to AtGA2ox6, two to AtGA2ox8, and four to AtGA2ox9, respectively (Fig. 1A). Among them, two homologues (Pos.gene27622 and Pos.gene52505) exhibited differential expression during chilling- and GA- induced EDR (Gai et al. 2013; Zhang et al. 2021). Based on high sequence similarity to AtGA2ox2 and AtGA2ox8, thus were named PsGA2ox2 and PsGA2ox8.1, respectively. The open reading frame (ORF) of *PsGA2ox2* was 996 bp, and that of *PsGA2ox8.1* was 990 bp, both containing the conserved 2OG-FeII_Oxy domain HMM matrix of GA2ox (Supplementary Fig. S1).

As known that 14 to 21 days after chilling (DAC) was the transition from endormancy to EDR, and 21 DAC was adequate for EDR in tree peony ‘Luhehong’ (Huang et al. 2008). GAs play an important role in the dormancy release of tree peony, and exogenous GAs significantly promotes EDR and budburst (Zhang et al. 2021). To determine which *PsGA2ox* members functioned in EDR and was potentially regulated by PsmiR159b, the expression patterns of *PsGA2ox2* and *PsGA2ox8.1* were detected using qPCR. Both genes were highly expressed at 0 DAC, followed by a dramatically decrease after 7 DAC. Subsequently, *PsGA2ox2* showed steady upregulation until 21 DAC, while *PsGA2ox8.1* expression remained relatively stable except for a slight increase at 21 DAC (Fig. 1B). These results indicated that both *PsGA2ox2* and *PsGA2ox8.1* functioned during chilling-induced EDR, consistent with the transcriptome data (Gai et al. 2013). Among them, *PsGA2ox2* exhibited an expression trend opposite to PsmiR159b during EDR (Zhang et al. 2024) (Fig. 1B), it was chosen for further investigation.

RNA in situ hybridization was performed to further investigate the spatial expression patterns of *PsGA2ox2* and PsmiR159b, and the results were consistent with that of qPCR, except for relative low signal intensity at 0 DAC (Fig. 1C). We speculated that buds of 0 DAC was in low tissue permeability associated with deep dormancy status. The tissue expression of *PsGA2ox2* was also evaluated at the early stage of flowering, as well as mixed buds of 0, 7, 14 and 21 DAC. *PsGA2ox2* transcripts showed high abundance in the sepal, bract, petal, and buds, while nearly undetectable in the stamen, carpel and leaf (Supplementary Fig. S2).

We further investigated the response of PsmiR159b and *PsGA2ox2* to exogenous GA_3_ using qPCR. PsmiR159b was significantly induced by GA_3_ treatment, and reached peak expression at 72 h (Fig. 1D). *PsGA2ox2* was also upregulated by exogenous GA_3_ at 12 h and then declined, whereas *PsGA2ox8.1* exhibited sustained increase after GA_3_ treatment for 12–72 h (Supplementary Fig. S3). Although both PsmiR159b and *PsGA2ox2* were induced by GA_3_, their expression trends diverged after GA_3_ feedings from 12 to 72 h, further suggesting a potential regulatory relationship (Fig. 1D).

Additionally, our recent research has demonstrated that PsmiR159b negatively regulates bud EDR by cleaving its target gene *PsMYB65* (Zhang et al. 2024). Here, to further investigate its regulatory network, we assessed the expression of *PsGA2ox2* in PsmiR159b-transgenic buds, which revealed that *PsGA2ox2* was dramatically depressed in OE-MiR159b buds and elevated in STTM159b buds (Fig. 1E). These expression patterns suggested a relationship between PsmiR159b and *PsGA2ox2*. The possibility of *PsGA2ox2* regulation by PsMYB65 was investigated using yeast one-hybrid (Y1H). The results showed no positive colonies on SD/-Leu/-Trp/-His medium with 3-AT, indicating that PsMYB65 could not bind the promoter of *PsGA2ox2*, despite the presence of the putative MYB-binding sites (Supplementary Fig. S4, S5). Consistently, the LUC assay confirmed that PsMYB65 did not enhance the activity of *PsGA2ox2* promoter (Supplementary Fig. S6). Furthermore, to rule out the possibility of regulation by the passenger strand (PsmiR159b*), target prediction using psRNAtarget software revealed no complementary relationship between PsmiR159b* and *PsGA2ox2* (Supplementary Fig. S7)*.* These finding suggested that *PsGA2ox2* was another target of PsmiR159b, and functioned in chilling-induced EDR of tree peony.

### *PsGA2ox*2 is a target of PsmiR159b

**Fig. 2:**
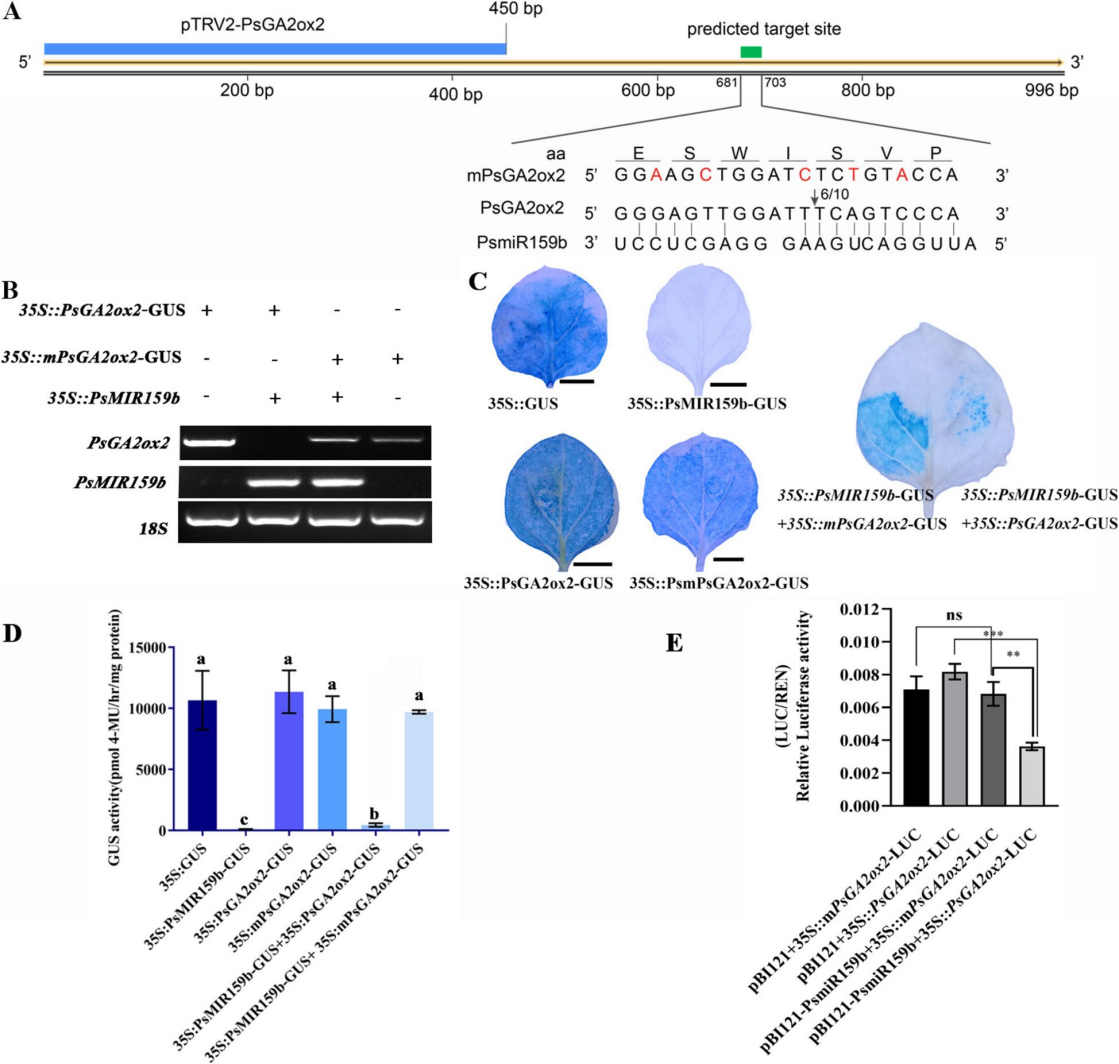
*PsGA2ox2* is directly cleaved by PsmiR159b**. A** A diagram of *PsGA2ox2* cDNA and RLM 5’-RACE assay revealed the PsmiR159b target site in *PsGA2ox2*. The positions of cleavage sites were indicated by arrow with the frequency of clones above. The green segment was the prediction target site of PsmiR159b in *PsGA2ox2*. Red fonts indicated synonymous mutant bases of *PsGA2ox2*. The blue segment was used to construct TRV2-*PsGA2ox2* vector in the VIGS experiment. **B** RT-PCR detection the expression of *PsGA2ox2* and PsmiR159b in transgenic tobacco leaves. Combination of *35S::PsGA2ox2-GUS* + *35S::PsMIR159b* and *35S::mPsGA2ox2-GUS* + *35S::PsMIR159b* were used to infect tobacco leaves, and those of *35S::PsGA2ox2-GUS* of *35S::mPsGA2ox2-GUS* alone were used for the control. The transcripts of *PsGA2ox2* and *PsMIR159b* were detected using RT-PCR. 18S rRNA was used as an internal reference. **C** and **D** GUS staining and GUS enzyme activity of leaves infected with different recombination vectors. Co-transformation of tobacco leaves with *PsMIR159b-*GUS + *PsGA2ox2*-GUS, *PsMIR159b-*GUS + *mPsGA2ox2*-GUS, respectively. GUS staining was observed histochemically (**C**). The GUS enzyme activity of leaves infected with different recombination vectors (**D**). Error bars indicated SE (*n* = 5). Different lowercase letters indicated statistically significant differences (one-way ANOVA, *P* < 0.05). **E** Dual-LUC assay validated the relationship between PsmiR159b and *PsGA2ox2*. Error bars indicated SE (*n* = 6). Asterisk indicated statistically significant differences (Student’s *t*-test, ** *P* < 0.01, *** *P* < 0.001)

Using RNAhybrid 2.2 software, a potential complementary site of PsmiR159b within the *PsGA2ox2* ORF (681–703 bp) was found (Fig. 2A). To verify the targeting relationship between PsmiR159b and *PsGA2ox2*, fusion vectors *PsMIR159b*-GUS and *PsGA2ox2*-GUS were constructed, along with *mPsGA2ox2*-GUS (containing synonymous mutations at the complementary sites) (Fig. 2A). GUS staining and activity assays revealed that leaves co-transformed with *PsMIR159b*-GUS and *PsGA2ox2*-GUS exhibited reduced staining and lower enzyme activity compared to those co-transformed with *PsMIR159b*-GUS and *mPsGA2ox2*-GUS (Fig. 2C, D).

To investigate the mechanism by which PsmiR159b modulated *PsGA2ox2*, the expression levels of *PsGA2ox2* in tobacco leaves co-infected with *35S::PsMIR159b* + *35S::PsGA2ox2* or *35S::PsMIR159b* + *35S::mPsGA2ox2* were analyzed by RT-PCR. The results showed that co-expression with PsmiR159b significantly decreased *PsGA2ox2* transcript levels. In contrast, *mPsGA2ox2* expression remained unchanged in leaves co-infected with *35S::PsMIR159b* + *35S::mPsGA2ox2* (Fig. 2B), indicating that PsmiR159b regulated *PsGA2ox2* expression primarily at the post-transcriptional level.

A dual-LUC assay was performed to further examine this interaction. *PsMIR159b* was inserted into pBI121, and *PsGA2ox2* into the pGreenII0800-miRNA vector, using *mPsGA2ox2* as a control. The fluorescence intensity dramatically declined when co-transforming *PsMIR159b* and *PsGA2ox2-LUC* compared to the control*,* whereas fluorescence intensity was unaffected when co-transformed *PsMIR159b* and *mPsGA2ox2-LUC* (Fig. 2E; Supplementary Fig. S8).

For further confirm the cleavage site, RLM5'-RACE was performed. Among 10 randomly sequenced clones, six clones exhibited cleavage site between nucleotides 693 (T) and 694 (T) of *PsGA2ox2* (Fig. 2A). Altogether, these results confirmed *PsGA2ox2* as a direct target of PsmiR159b in tree peony.

### GA contents during chilling-induced EDR

**Fig. 3:**
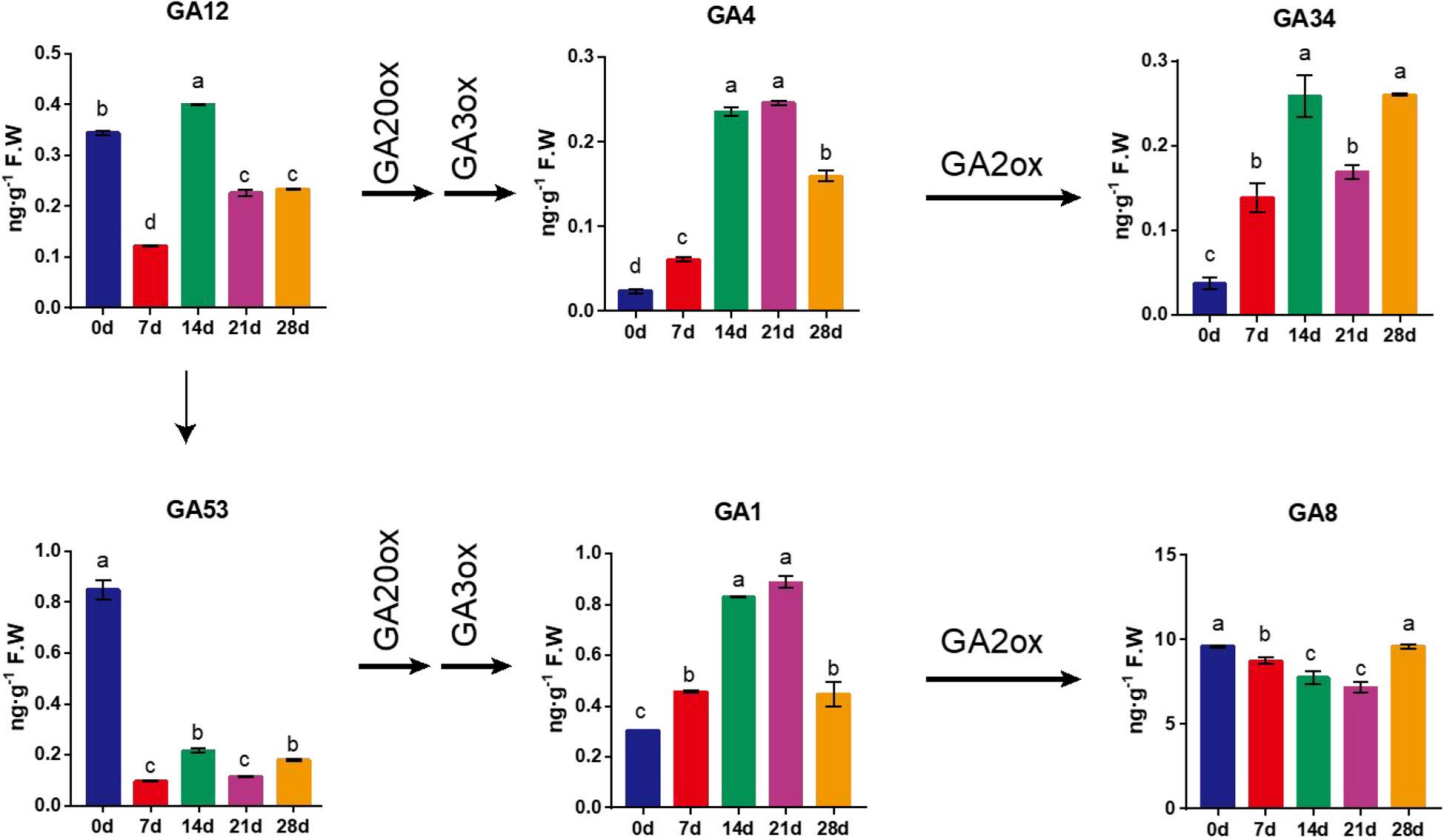
The GA contents during chilling-induced EDR in tree peony. The GA contents were determined by LC–MS/MS. Error bars indicated SE (*n* = 3). Arrows showed the enzymes in charge of the conversion. Different lowercase letters indicated statistically significant differences (one-way ANOVA, *P* < 0.05)

As known, GA2ox catalyzes the conversion of bioactive GA_1_ and GA_4_ into their catabolites GA_8_ and GA_34_, respectively (Kurepin et al. 2013). Therefore, we monitored the changes of GA analogues during EDR in tree peony. Bioactive GA precursors, such as GA_12_, peaked at 14 DAC, while GA_53_ exhibited its highest concentration in non-chilled buds. Notably, prolonged chilling significantly elevated the levels of bioactive GAs, with GA_1_ and GA_4_ reaching peak at 14 to 21 DAC, corresponding to critical stages of dormancy release. Concurrently, GA_34_, a product of GA2ox catalysis, increased significantly, while GA_8_ levels decreased from 0 to 21 DAC. GA_1_ maintained higher concentrations than GA_4_ throughout the process, consistent with the observation that most higher plants primarily utilize GA_1_ as the biologically active endogenous GA. Correspondingly, GA_8_, produced from GA_1_ via GA2ox, showed higher prevalence than GA_34_ (Fig. 3). These findings suggested that GA_34_ might be a primary product of PsGA2ox2, whereas GA_8_ was likely produced from other PsGA2ox member.

### *PsGA2ox2* maintains bud dormancy

**Fig. 4:**
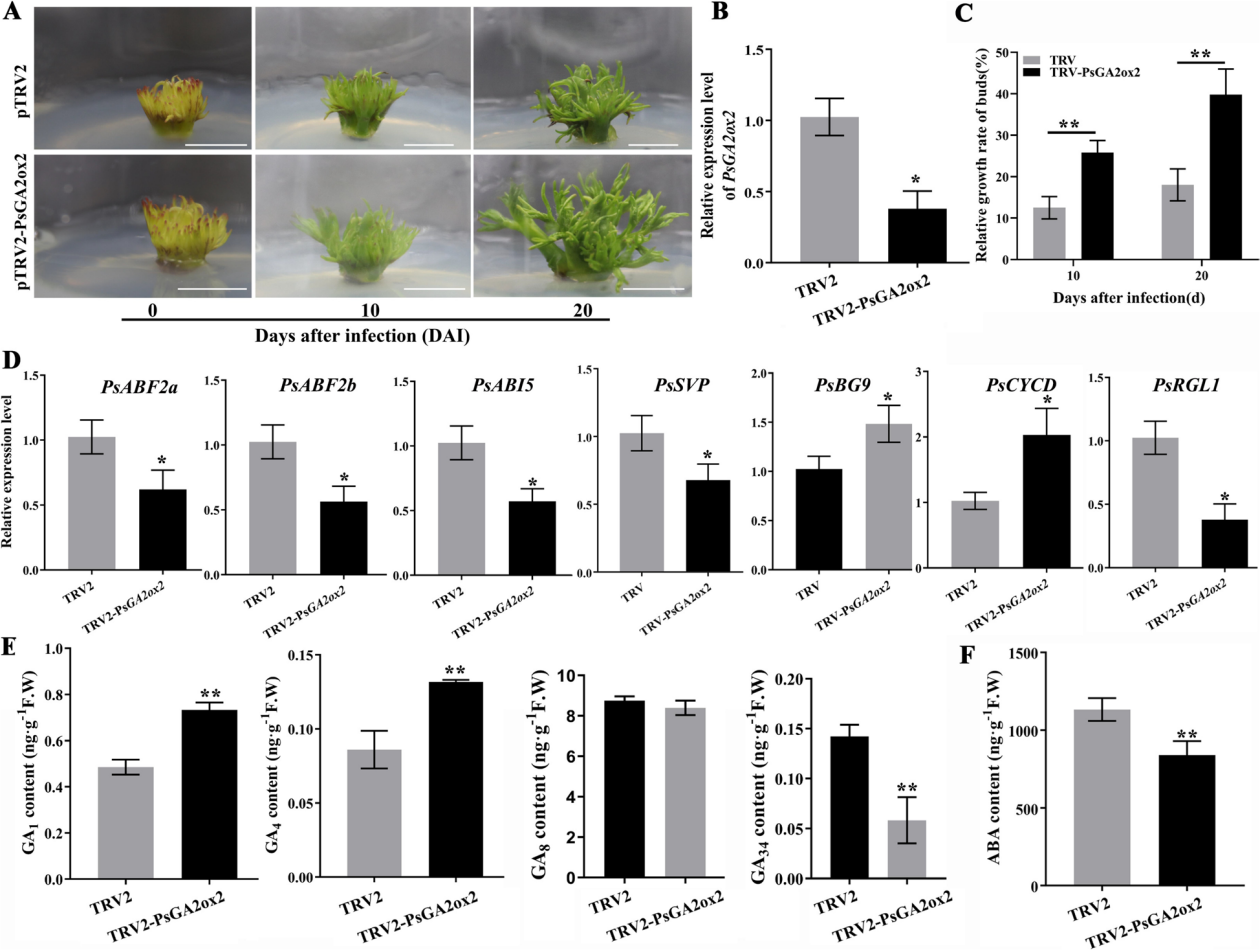
Silencing of *PsGA2ox2* accelerated bud EDR in tree peony. **A** Tree peony buds phenotype after silencing of *PsGA2ox2* at 0, 10 and 20 DAI. Buds infected by TRV1 + TRV2 served as mock. Bar = 1 cm. **B** Relative expression levels of *PsGA2ox2* in *PsGA2ox2*-silenced buds at 7 DAI. Error bars indicated SE (*n* = 3). Asterisks indicated statistically significant differences (Student’s *t*-test, *P < 0.05). **C** The relative growth ratio of TRV2-*PsGA2ox2* buds at 10 and 20 DAI. Error bars indicated SE (*n* = 9). Asterisks indicated statistically significant differences (Student’s *t*-test, **P < 0.01). **D** The relative expression levels of genes associated with EDR in *PsGA2ox2-*silenced buds at 7 DAI. Asterisks indicated the significant differences (Student’s *t*-test, *P < 0.05). **E** and **F** The contents of GAs (**E**) and ABA (**F**) in TRV2-*PsGA2ox2* buds. Error bars indicated SE (*n* = 3). Asterisks indicated statistically significant differences (Student’s *t*-test, **P < 0.01)

To investigate the role of *PsGA2ox2* during EDR in tree peony, *PsGA2ox2* was silenced in 7d-chilled buds using a virus-induced gene silencing (VIGS) method (Zhang et al. 2023). A TRV2-*PsGA2ox2* vector was constructed, and *A. tumefaciens* EHA105 containing TRV1 + TRV2-*PsGA2ox2* was used to infect the buds, with combination of TRV1 + TRV2 serving as a control (Fig. 4A). After 7 days, silencing efficiency of *PsGA2ox2* was identified using qPCR. The results showed that the expression level of *PsGA2ox2* in TRV1 + TRV2-*PsGA2ox2* decreased approximately 50% compared with the control (Fig. 4B). Bud dormancy in woody plants is triggered by SD and low temperatures, leading to the growth cessation of shoot apical meristem (SAM) (Bohlenius et al. 2006). SAM starts developing while the bud dormancy is released, so the relative growth rate serves as an indicator of bud dormancy release. Measurements of bud height at 10 and 20 days after infection (DAI) revealed that buds after silencing of *PsGA2ox2* grew faster than the control (Fig. 4A and C).

Since GA antagonizes ABA, the ABA response transcription factor genes (*PsABF2a*, *PsABF2b*, and *PsABI5*) were analyzed during chilling-induced EDR (Supplementary Fig. S9-S10). In TRV2-*PsGA2ox2* buds, *PsABF2a*, *PsABF2b*, *PsABI5*, *PsSVP* and *PsRGL1* were significantly downregulated, whereas marker genes associated with EDR including *PsBG9* and *PsCYCD* showed upregulation (Fig. 4D). ABA content decreased in TRV2-*PsGA2ox2* buds (Fig. 4F).

Endogenous GA levels were measured, which showed that the contents of the bioactive GAs, GA_1_ and GA_4_, increased significantly in TRV2-*PsGA2ox2* buds, whereas GA_34_ levels declined substantially (Fig. 4E). The result was consistent with that in *Populus* (Gou et al. 2011). Similarly, OE-*MIR159b* dramatically enhanced the contents of GA_1_ and GA_4_ and decreased GA_34_ levels, whereas silencing *PsMIR159b* reduced GA_4_ contents and elevated GA_34_ levels (Supplementary Fig. S11). Thus, *PsGA2ox2* negatively regulated EDR. PsmiR159b and *PsGA2ox2* displayed opposing effects on GA analogues, which further supported that *PsGA2ox2* was targeted by PsmiR159b.

### Ectopic expression of *PsGA2ox2* inhibits seed germination in Arabidopsis

**Fig. 5:**
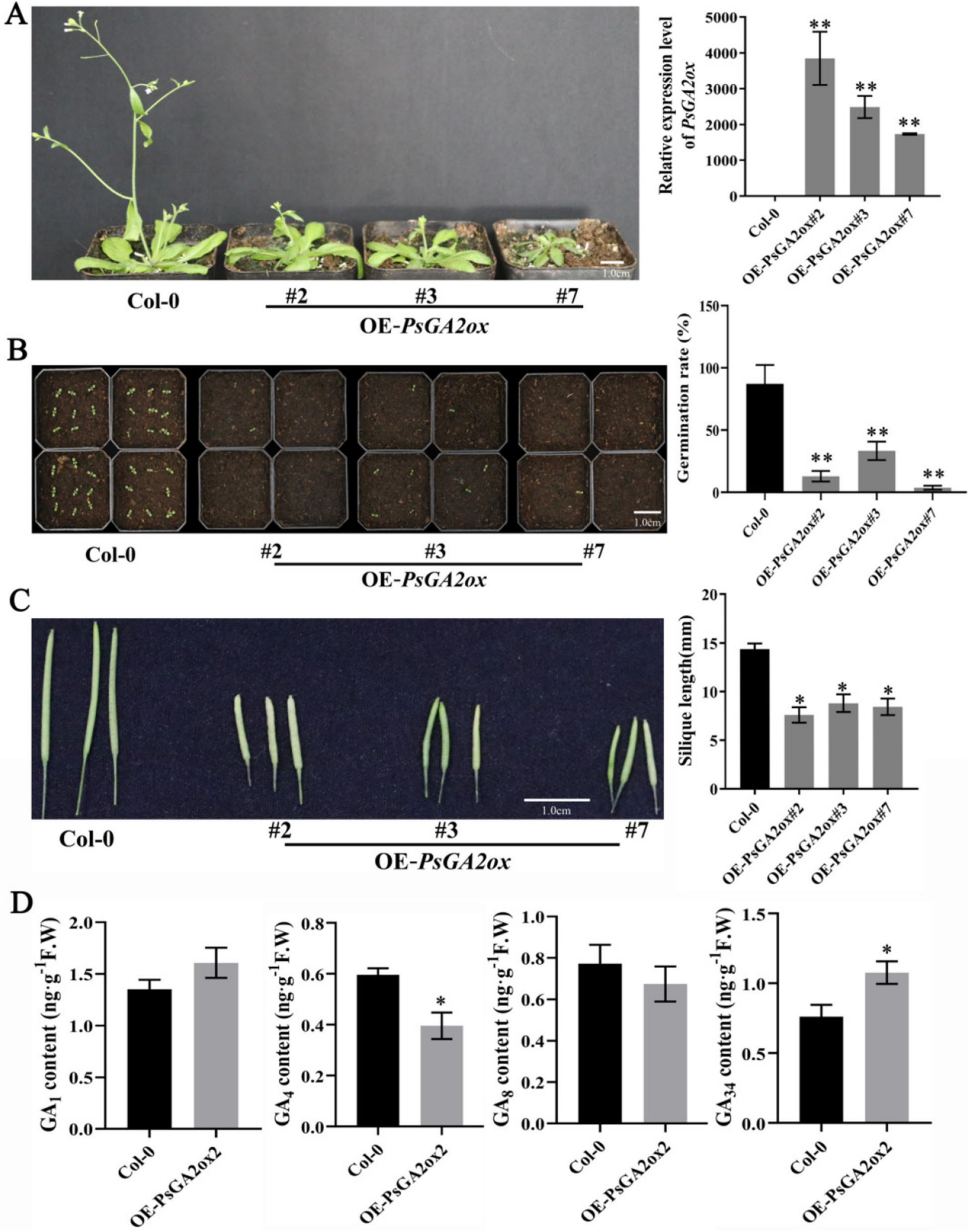
The phenotype of ectopic expression *PsGA2ox2* in *Arabidopsis thaliana*. **A** The phenotype of OE-*PsGA2ox2* Arabidopsis. Transgenic Arabidopsis was screened on 1/2 MS medium with 50 mg/mL kanamycin. T_3_ generation was used to observe phenotype at 35 days after germination. The expression of *PsGA2ox2* in transgenic Arabidopsis detected by qPCR (right). **B** Ectopic overexpression of *PsGA2ox2* delayed seed germination in Arabidopsis. The transgenic seeds were cultured in a greenhouse for 7 d to take photoes. The seed germination rate of OE-*PsGA2ox2* was shown on the right. Col-0: pBI121; OE-*PsGA2ox2*: overexpression of *PsGA2ox2*. **C** Phenotype of siliques of OE-*PsGA2ox2*. The silique lengths were shown on the right. Col-0: pBI121; OE-*PsGA2ox2*: overexpression of *PsGA2ox2*. **D** The contents of GA analogs in OE-*PsGA2ox2* lines. Error bars indicated SE (*n* = 3). Asterisks (**A**-**D**) indicated statistically significant differences (Student’s *t*-test, *P < 0.05, **P < 0.01)

To further investigate the potential function of *PsGA2ox2*, T_3_ transgenic Arabidopsis lines heterologously expressing *PsGA2ox2* were generated under the control of the CaMV 35S promoter, and three independent OE-*PsGA2ox2* lines were analyzed (Fig. 5). Compared with the control, the seed germination rate of OE-*PsGA2ox2* lines significantly decreased after sowing for 7 days (Fig. 5B). The OE-*PsGA2ox2* lines exhibited slower vegetative growth and a considerable decrease in plant height (Fig. 5A). Furthermore, their siliques were notably shorter in length (Fig. 5C). These findings indicated that heterologous overexpression of *PsGA2ox2* affected both vegetative and reproductive growth of Arabidopsis. Analysis of GA analogs revealed that in OE-*PsGA2ox2* lines, bioactive GA_4_ levels were significantly decreased, while GA_34_ levels were drastically elevated (Fig. 5D). In contrast, no significant difference was detected in GA_1_ and GA_8_ contents between Col-0 and OE-*PsGA2ox2*.

### Spatio-temporal expression of *PsMYB65* and *PsGA2ox2*

**Fig. 6:**
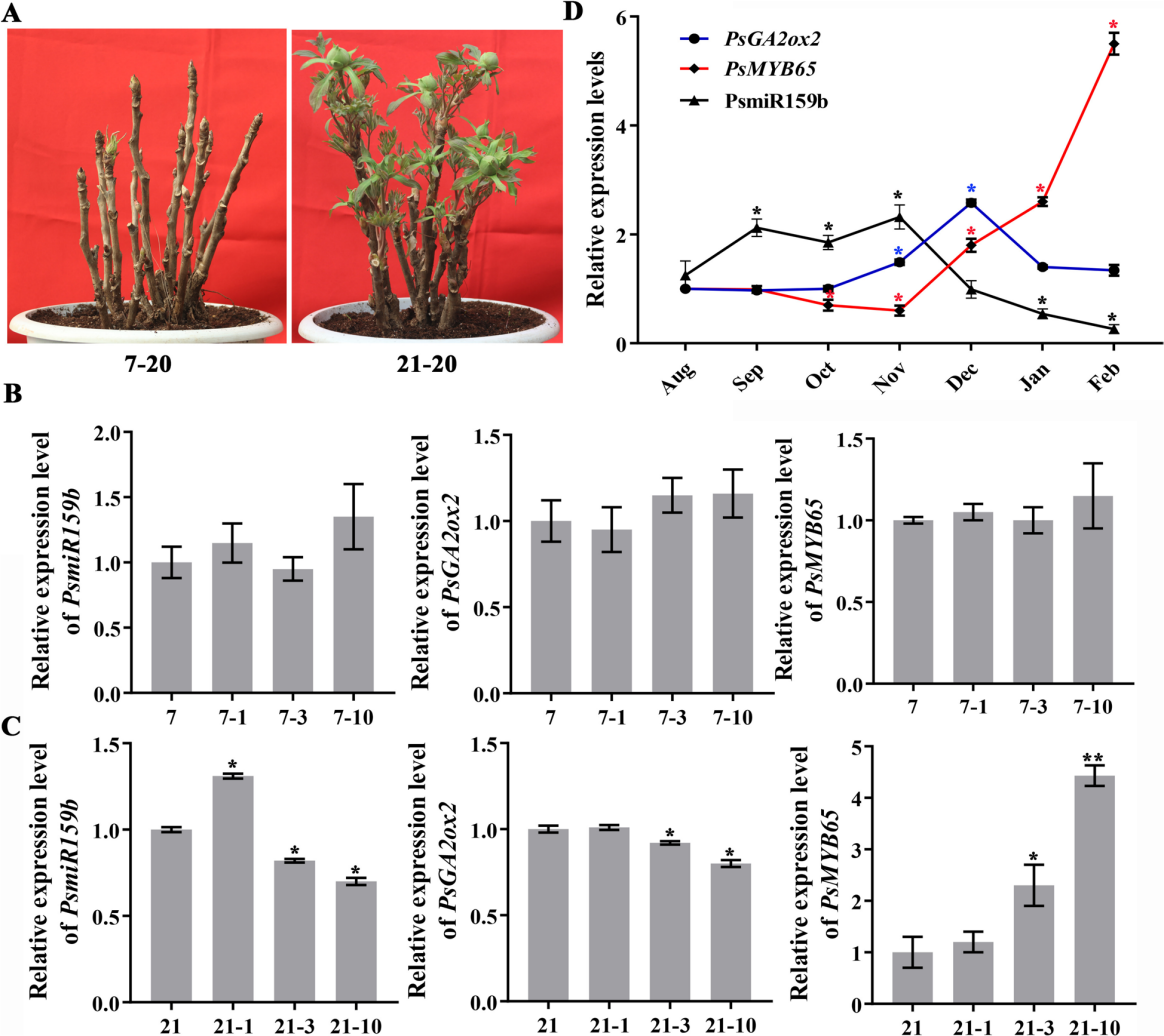
Spatio-temporal expression patterns of PsmiR159b*, PsGA2ox2* and *PsMYB65* during bud endodormancy induction, EDR, and budburst. **A** Morphology of 7 DAC (left) or 21 DAC (right) -chilled tree peony after being transferred to greenhouse for 20 days. **B**, **C** Expression patterns of PsmiR159b, *PsGA2ox2*, and *PsMYB65* in 7 d (**B**) or 21 d (**C**) -chilled tree peony after being moved into the greenhouse for 1, 3, and 10 days. **D** The expression patterns of PsmiR159b, *PsGA2ox2*, and *PsMYB65* during the whole bud dormant stage under natural conditions. Error bars indicated SE (*n* = 3). Asterisks indicated statistically significant differences (Student’s *t*-test, *P < 0.05, **P < 0.01)

Our recent study demonstrated that PsmiR159b targets *PsMYB65*, and the PsmiR159b-*PsMYB65* module participates in the regulation of EDR in tree peony (Zhang et al. 2024). To further analyze the selectivity of PsmiR159b toward *PsGA2ox2* and *PsMYB65* during tree peony EDR and budburst, their spatio-temporal expression patterns were evaluated. When buds were transferred to the greenhouse after 7 DAC, no significant changes were observed in the expression of *PsGA2ox2* and *PsMYB65*, along with a stationary PsmiR159b (Fig. 6B). However, when plants were moved to the greenhouse after 21 DAC, the transcription levels of *PsGA2ox2* were continuously downregulated, whereas *PsMYB65* were significantly upregulated. Meanwhile, PsmiR159b was upregulated after 1 d followed by a significant decrease until 10 d. The upregulation of *PsMYB65* might be due to the decrease in PsmiR159b expression and the accumulation of bioactive GAs induced by prolonged chilling (Fig. 6C), while *PsGA2ox2* lost its sensitivity to PsmiR159b in a drastic temperature changes condition.

*PsGA2ox2* and *PsMYB65* expressions were also monitored during the dormancy transition under natural conditions. *PsGA2ox2* expression level gradually increased in the dormancy establishment stage (Fig. 6D, Oct.-Nov.), and reached to peak in the EDR stage (Fig. 6D, Dec.), followed by downregulation in the budbreak stage (Fig. 6D, Dec.-Feb.). In contrast, *PsMYB65* expression decreased gradually in the dormancy establishment stage (Fig. 6B, Oct.-Nov.), and significantly increased at the budbreak stage (Fig. 6D, Dec.-Feb.), indicating *PsMYB65*’s role in EDR (Fig. 6D). The initial increase in *PsMYB65* expression before endodormancy might be relate to flower bud organ development (Fig. 6D). Overall, *PsGA2ox2* and *PsMYB65* presented similar expression patterns during bud formation to bud EDR, particularly under PsmiR159b regulation.

## Discussion

Endodormancy in tree peony affects its flowering time and flower quality, thereby seriously limiting the development of tree peony industry. Previous studies have demonstrated that bioactive GAs accelerate the EDR and flowering in tree peony (Zhang et al. 2021; Gao et al. 2023). However, the regulatory mechanism of GA signaling on EDR is poorly described. PsmiR159b is abundant in dormant tree peony buds, and the miR159b-*MYB65* module regulates budbreak by modulating the cell cycle (Zhang et al. 2024). In this study, we identified a novel target of miR159b, *PsGA2ox2*, a GA catabolic gene. Silencing of *PsGA2ox2* accelerated EDR and budburst. miR159b-*PsGA2ox2* regulated the contents of bioactive and deactive GAs during chilling duration. Our findings revealed a novel regulation pathway of GA catabolism and bud EDR in tree peony.

### *PsGA2ox2* maintains bud endodormancy in tree peony

To survive in cold winter, deciduous perennial plants have evolved a physiological mechanism known as bud dormancy. During the process, plants also maintain the ability to awaken themselves and prepare for the resumption of growth, which is typically triggered by chilling accumulation and external cues such as exogenous bioactive GAs. As chilling accumulates, the content of bioactive GAs increases, while that of ABA decreases (Tylewicz et al. 2018; Zhang et al. 2020), thereby influencing the timing and progression of dormancy. In tree peony, prolonged chilling continuously increases the content of bioactive GAs (Fig. 3), and reduces ABA content (Zhang et al. 2020). Furthermore, exogenous GAs accelerate EDR, indicating that GAs play a vital role in bud dormancy-activity transition in tree peony.

GA biosynthesis undergoes tight regulation via feedback mechanisms, and bioactive GAs can be metabolized to GA_34_ and GA_8_ through GA2oxs. Overexpression of *GA2ox* reduces bioactive GA content by converting the precursors such as GA_12_ and GA_53_ (Varbanova et al. 2007; Zhu et al. 2023). GA2ox has been implicated in the induction and maintenance of seed and bud dormancy (Mauriat et al. 2011). In *populus*, overexpression of *GA2ox* leads to growth inhibition under drought and SD conditions (Zawaski et al. 2014). Similarly, *PavGA2ox-2L* overexpression results in decrease of plant height, lower seed germination rate, and delayed flowering in *Arabidopsis thaliana* (Liu et al. 2022a, b). Our study demonstrated that ecotopic expression of *PsGA2ox2* also led to draft phenotype and inhibited seed germination (Fig. 5), suggesting a conserved function of GA2oxs among plant species. Conversely, silencing *PsGA2ox2* significantly promoted the budbreak and bud growth in tree peony (Fig. 4), which revealed a function of maintaining dormancy.

Accordingly, the contents of GA_1_ and GA_4_ significantly increased in *PsGA2ox2-*silenced buds (Fig. 4), indicating that *PsGA2ox2* inhibited EDR by reducing bioactive GA levels. In tree peony, GA_34_, a deactivation product of GA_4_, was upregulated by chilling duration with a maximum at 14 and 28 DAC (Fig. 3), which was closely correlated with the expression pattern of *PsGA2ox2* (Fig. 1). In contrast, GA_8_, a deactivation product of GA_1_, declined until 21 DAC, and its associated gene *PsGA2ox8.1* was dramatically downregulated by chilling accumulations (Fig. 1). We hypothesized that *PsGA2ox2* might be more sensitive to GA_4_. High expression of *PsGA2ox2* inhibited the accumulation of bioactive GAs, thereby delaying bud EDR and budburst. These findings demonstrated that *PsGA2ox2* maintained bud dormancy through the catabolism of bioactive GAs in tree peony buds.

In addition, the expression of ABA-related genes in *PsGA2ox2*-silenced buds suggested the involvement of ABA signaling in the process (Fig. 4). ABA serves as a key regulator of bud dormancy (Pan et al. 2021). ABF2, ABI5 function as essential components of the ABA pathway, while SVP acts as a feedforward regulator of ABA biosynthesis (Singh et al. 2018; Tylewicz et al. 2018), consequently inducing and maintaining bud dormancy. In *PsGA2ox2*-silenced buds, the decrease of *PsABF2a*, *PsABF2a*, *PsABI5*, and *PsSVP*, alongside the increase of bioactive GA levels (Fig. 4), suggested that GA accumulation suppresses ABA biosynthesis and signal transduction, supporting our previous findings (Zhang et al. 2021). ABA and GA antagonistically mediate several biological processes. For instance, in *Populus*, ABA inhibits GA biosynthesis by inhibiting *GA20ox* by SVL (Singh et al. 2018; Tylewicz et al. 2018). In ‘Suli’ pear, ABA inhibits *GA20ox* by downregulating GAST1, thereby delaying bud EDR (Yang et al. 2019), while PpyABF3 activates the expression of *PpyGA2ox1* to reduce bioactive GAs (Yang et al. 2023). However, the mechanism by which GA antagonizes ABA remains largely unexplored and requires further investigation.

### *PsGA2ox2* is a novel target of PsmiR159b

miR159 is strongly conserved and highly abundant microRNA throughout the plant kingdom, known for targeting a class of GA response genes called GAMYB or GAMYB-like via highly conserved miR159-binding sites, regulating various developmental processes, including vegetative, anther, seed development, and flowering (Millar et al. 2019). In tree peony, PsmiR159s constitute the most abundant small RNA species, with PsmiR159b functioning as a major regulator of bud EDR (Zhang et al. 2024). The PsmiR159b-*PsMYB65* module mediates the dormancy-growth transition (Zhang et al. 2024). Beyond GAMYB-like genes, miR159 targets additional genes. For example, in tomato, a gene with a NOZZLE-like domain, *SGN-U567133*, represents a novel miR159 target (Buxdorf et al. 2010). In Arabidopsis, the *OPT1* gene undergoes miR159-mediated cleavage (Schwab et al. 2005). In rose, miR159 targets *Cytokinin Oxidase*/*Dehydrogenase 6* (*CKX6*), modulating petal size via cytokinin catabolism (Jing et al. 2023).

In this study, *PsGA2ox2* was identified as a potential target of miR159 in the dormant bud transcriptome of tree peony. The expression trends of PsmiR159b and *PsGA2ox2* showed inverse correlation during chilling- and GA- induced EDR processes using qPCR and in situ hybridization (Fig. 1). Additionally, genetic analysis, GUS staining, and GUS enzyme activity confirmed that PsmiR159b targeted *PsGA2ox2* (Fig. 2). These results establish *PsGA2ox2* as a novel target of PsmiR159b in dormant buds of tree peony.

GA2ox acts as a deactivating enzyme of bioactive GA_1_ and GA_4_, thus playing a crucial role in GA homeostasis. Recent studies have discovered several transcription factors modulating the expression of *GA2oxs*. In soybean, an HD-ZIP III transcription factor, ATHB14-LIKE, binds to the promoter of *GmGA2ox2* and activates its expression (Zhao et al. 2022). In *Sorghum,* ABI4 and ABI5 directly activate the transcription of *GA2ox* (Renata et al. 2013). VvSVP1 binds to the promoter of *VvGA2ox3* to regulate GA contents and bud EDR in grape (Li et al. 2025). Currently, no microRNA has been identified to regulate the expression of *GA2ox*. PsmiR159b-*PsGA2ox2* represents a novel pathway in GA homeostasis regulation in tree peony.

### PsmiR159b serves as a bifunctional regulator of GA response

Up to now, we have identified two distinct targets of PsmiR159b, *PsGA2ox2* and *PsMYB65*. Intriguingly, these two genes play diverse functions in dormancy regulation, and *PsGA2ox2* maintains dormancy, whereas *PsMYB65* accelerates EDR and budbreak. Therefore, we want to explore how PsmiR159b discriminates between *PsGA2ox2* and *PsMYB65* throughout dormancy progression. Expression patterns of *PsGA2ox2* and *PsMYB65* from budset (Aug.) to budbreak (Feb.) indicated that both genes exhibited similar trends from Nov. to Dec. Accordingly, PsmiR159b displayed an opposite pattern in the same timespan (Fig. 6D). Spatially, *PsMYB65* is highly expressed in the stamen, carpel, and bract of early flowers, as well as in dormant buds (Zhang et al. 2024), and *PsGA2ox2* is preferentially expressed in the bract, sepal, petal of early flowers, and the dormant buds (Fig. S2). Additionally, PsmiR159b is also abundant in stamen, carpel, and bract of early flower, and the dormant buds (Zhang et al. 2024). This overlap in the dormant buds suggested that PsmiR159b may function in concert with both PsGA20ox2 and PsMYB65 to modulate GA homeostasis and EDR.

**Fig. 7:**
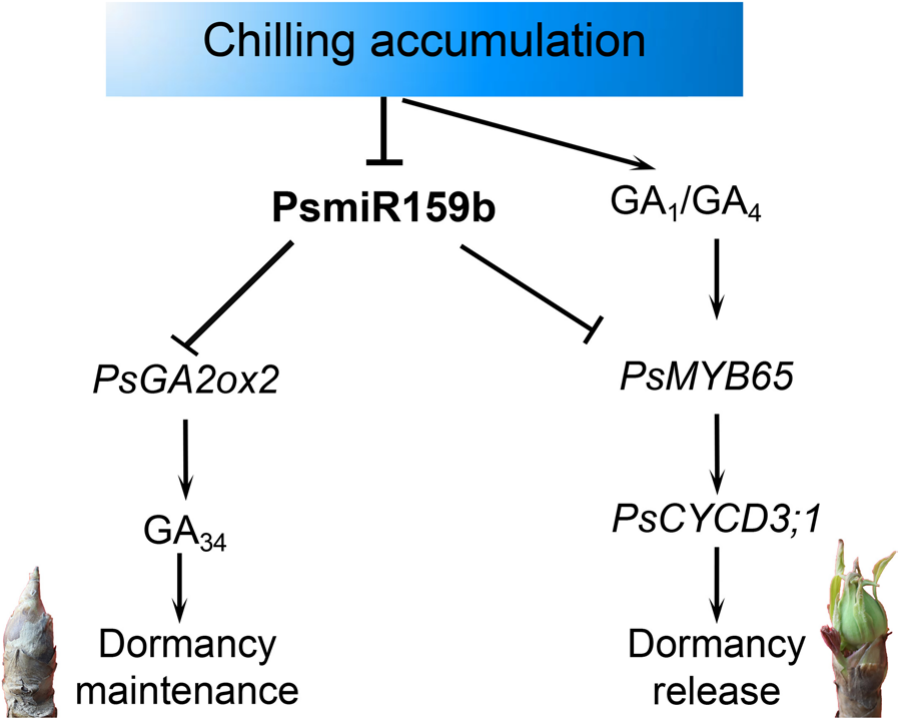
The model of PsmiR159b regulating *PsGA2ox* and *PsMYB65* during chilling-induced EDR. PsGA2ox2 deactived bioactive GAs to inhibit bud EDR and maintain dormancy state. PsmiR159b served as a precise adaptor to regulate the expression levels of *PsMYB65* and *PsGA2ox2*, and participated in regulating bioactive GA content and GA response during chilling-induced EDR

PsmiR159b acted as a bifunctional regulator of GA homeostasis in dormant tree peony buds by targeting two antagonistic nodes in the GA pathway. As known, the increasing bioactive GAs are feedback-regulated to maintain GA homeostasis, partially through deactivation by GA2ox and other enzymes. Additionally, the presence of PsmiR159b might balance bioactive GA contents and GA responses according to our results. As chilling prolonged, with increasing bioactive GAs levels, the expression of PsmiR159b decreases, allowing *PsMYB65* levels to increase to promote GA response and subsequent cell proliferation (Zhang et al. 2024). Simultaneously, *PsGA2ox2* expression increased to deactivate a portion of the bioactive GAs, thereby delaying bud EDR. These findings suggested that PsmiR159b functioned as a novel bifunctional regulator in the GA pathway during EDR (Fig. 7).

In conclusion, this study identified *PsGA2ox2* as a novel target of miR159b in tree peony, demonstrating its role in deactivating bioactive GAs to inhibit bud EDR. PsmiR159b acted as an precise adaptor regulating bioactive GA levels and GA responses during chilling-induced EDR. These findings enhance the understanding of GA regulation and provide new insights into the molecular mechanisms underlying bud dormancy in tree peony.

## Materials and methods

### Plant materials and treatment

Four-year-old tree peony (*P. suffruticosa* ‘Luhehong’) were potted and subjected to chilling treatment at 4℃ in a dark refrigerator as described previously (Xin et al. 2019). Apical buds were collected at 0, 7, 14, 21 and 28 days of DAC. An equal number of unsampled plants were also moved to the greenhouse to assess dormancy status, as previously described (Zhang et al. 2020).

A total of 90 plants after 7 DAC were transferred to a greenhouse (25℃/18℃, 8 h light/16 h dark), and 200 mg·L^−1^ GA_3_ (G8910, Solarbo, China) was applied, with double-distilled water serving as a mock. Then, the apical buds were harvested after 0, 12, 24, and 72 h of GA_3_ treatment.

Naturally grown buds were sampled monthly on the 15th from August (budset) to the following February (budbreak). All collected apical buds were immediately frozen in liquid nitrogen and stored at −80℃ for subsequent analysis. Each treatment included three replicates, with 3 plants per replicate.

*PsMIR159b*-overexpressing buds (OE-*PsMIR159b*) and *PsMIR159b*-silenced buds (STTM159b) were obtained previously (Zhang et al. 2024).

### Sequence processing and phylogenetic analysis

Arabidopsis GA2ox protein sequences were obtained from TAIR (https://www.arabidopsis.org/), and published GA2ox homologs were obtained from NCBI. Tree peony GA2ox candidate sequences were screened from our transcriptome and genome databases (Zhang et al. 2021; Yuan et al. 2022). Multiple sequence alignment and neighbor-joining phylogenetic tree construction were performed in MEGA 11.0 software. The structure of *PsGA2ox* was illustrated using IBS 1.0.3, and targets of PsmiR159b were predicted using psRNATarget (http://plantgrn.noble.org/psRNATarget/).

### RNA extraction and gene expression analysis

Total RNA was extracted using the MiniBEST Plant RNA Extraction Kit (Takara, Dalian, China). First-strand cDNA was synthesized using the Evo M-MLV Mix Kit (Accuraye Biotechnology, Changsha, China). Quantitative Real-time PCR (qPCR) was conducted using SYBR Green Premix Pro Taq HS qPCR Kit (Accurate Biology, Changsha, China). The gene expression levels were analyzed using the 2^−ΔΔCt^ method. The *PsActin* gene was used as an internal control for normalization (Zhang et al. 2023). The primers were listed in Supplementary Table S1.

### qPCR for miRNA

The 200 μg total RNA was reverse-transcribed with an miRNA-specific stem-loop forward primer using the Vazyme miRNA 1st Strand cDNA Synthesis Kit (by stem-loop) (Vazyme, MR101-01, Nanjing, China). qPCR was performed using the previous described method (Zhang et al. 2024). *PsU6* was used as an internal control for normalization. The primers used were listed in Supplementary Table S1.

### RNA in situ hybridization

In situ hybridization was performed using DIG High Prime DNA Labelling and Detection Starter Kit (Roche, USA) as described previously (Zhang et al. 2015). Briefly, fresh buds after chilling treatments were fixed in FAA solution and cut into 8 μm thickness following embedding in paraffin. Specific probes were designed based on non-conserved region of *PsGA2ox2*, and probe labeling was performed using the RNA Labeling Kit (Roche, USA). The primer pairs for the sense probe of *PsGA2ox2* was GA2ox-senseF and GA2ox-senseR, and those for the antisense probe were GA2ox-antiF and GA2ox-antiR. The sense probe of PsmiR159b was 5’-GAATTGTAATACGACACACTATAGTTTGGATTGAAGGGAGCTCTA-3’, and the antisense was 5’-GAATTGTAATACGACACACTATAGATTGGATTGAAGGGAGCTCCT-3’. The primers used were listed in Supplementary Table S1. Hybridization signals were visualized on a Leica TCS-SP2 microscope.

### RLM 5’-RACE

To survey the PsmiR159b cleavage site on *PsGA2ox2* transcripts, RNA ligase-mediated 5’- RACE (RLM 5’-RACE) assay was performed using the HiScript-TS 5'/3' RACE Kit (Vazyme, Nanjing, China). In brief, the total RNA from chilled buds for 0, 7, 14, 21, and 28 d was attached to 5’RACE adapter using T_4_ RNA ligase. The ligated RNA was used to synthesize cDNA using M-MLV reverse transcriptase. The PCR products were purified and cloned into the 18-T vector, and ten randomly selected single clones were sequenced to determine the cleavage site. The primers used in this assay were listed in Supplementary Table S1.

### Target relationship identification of PsmiR159b to *PsGA2ox2*

To verify the target relationship between PsmiR159b and *PsGA2ox2*, the precursor of PsmiR159b, *PsMIR159b,* was amplified, and then *PsMIR159b* and *PsGA2ox2* were fused with GUS in the pBI121 vector to construct *35S*::*PsGA2ox*-*GUS* and *35S*::*PsMIR159b*-*GUS* vectors, with *35S*::*GUS* serving as a positive control. Subsequently, GUS staining and enzyme activity were analyzed. The predicted target site of *PsGA2ox2*, 5’-GGGAGTTGGATTTCAGTCCA-3’, was synonymously mutated to 5’-GGAAGCTGGATCTCTGTACCA-3’ and fused with GUS to construct the *mPsGA2ox2*-GUS vector. These recombinant vectors were introduced into the *Agrobacterium tumefaciens* strain GV3101. The transformation method in *Nicotiana benthamiana* followed the previously reported protocol (Norkunas et al. 2018). GUS staining and GUS activity analysis were performed using the method described by Jefferson et al. (1987).

### Virus Induced Gene Silencing (VIGS)

VIGS was performed to identify the function of *PsGA2ox2* following established protocols (Gao et al. 2023). In briefly, a specific 450 bp fragment of *PsGA2ox2* was inserted into the TRV2 vector to construct TRV2-*PsGA2ox2* (Fig. 2A). The primers using in this assay were listed in Supplementary Table S1. The TRV1, TRV2, and TRV2-*PsGA2ox* were transformed into *A. tumefaciens* strain EHA105. The recombinant colonies were cultured in LB liquid medium containing 50 mg·mL^−1^ kanamycin and 50 mg·mL^−1^ rifampicin until reaching an OD_600_ of 0.6–0.8. The *A. tumefaciens* strain cells were collected and resuspended in infiltration buffer (10 mM MgCl_2_, 50 mM Acetosyringone, 10 mM 2-Morpholinoethanesulphonic acid, pH 5.6). The infection solution containing equal volumes of TRV1 + TRV2 and TRV1 + TRV2-*PsGA2ox2* were placed in the dark for 3 h before use.

For VIGS implementation, buds treated for 7 DAC were immersed in infection buffer using a vacuum pump at −0.07 MPa, maintained for 10 min, with two repetitions. Subsequently, the buds were planted in 1/2 MS culture medium and maintained in a light culture chamber at 22℃ under 16/8 h light/dark conditions (Gao et al. 2023). The primers used for vectors construction and genes expression analysis were listed in Supplementary Table S1.

The morphological changes of buds 0, 10 and 20 DAI were evaluated according to the method of Zhang et al. (2024).

### Arabidopsis transformation and germination assays

To obtain *PsGA2ox2* overexpression lines, the *PsGA2ox2* ORF was amplified and cloned into the pBI121 vector driven by the 35S promoter. The recombinant vector pBI121-*PsGA2ox2* was introduced into *A. tumefaciens* strain GV3101 and then transformed into Arabidopsis using the floral dip method (Clough and Bent 2010). Positive transformants were selected on 1/2 MS medium containing 50 mg·mL^−1^ kanamycin and confirmed by PCR. T_3_-generation plants were utilized for seed germination analysis following established protocols (Chen et al. 2020). In briefly, seeds of empty pBI121 and OE-*PsGA2ox2* lines were cultivated in a greenhouse for 7 d to assess germination rate. For each line, at least 36 seeds per replicate (three biological replicates) were sown on soil.

### Y1H assay

Promoter regions of *PsGA2ox2* were amplified from tree peony genomic DNA (extracted using the Hi-DNAsecure Plant Kit, TIANGEN) (Yuan et al. 2022) and analyzed for putative MYB‐binding motifs using PlantCARE software. Four overlapping fragments covering all predicted cis-elements, as well as the full-length promoter, were cloned upstream of the HIS3 reporter in pHIS2.1. The *PsMYB65* ORF was fused into the pGADT7 vector to conduct Y1H assay. These constructs were then transformed into yeast Y187 competent cells, and 3-AT was used to suppress the background expression. Transformants were screened on SD/-Trp/-Leu and SD/-Trp/-His/-Leu media in turn. All primers for yeast assays were listed in Supplemental Table S1.

### Dual-luciferase (LUC) assay

To determine the possible effects of PsMYB65 on *PsGA2ox2*, the ORF of *PsMYB65* was inserted into pGreenII0029 62-SK to generate the effector, while promoter fragments of *PsGA2ox2* were inserted into the pGreenII 0800-LUC vector to construct reporters. These effector and reporters were then co-transformed into *N. benthamiana* leaves.

To verify the relationship between PsmiR159b and *PsGA2ox2*, the precursor of PsmiR159b, *PsMIR159b*, was cloned into pGreenII0029 62-SK, and *PsGA2ox2* was integrated into the pGreenII 0800-miRNA vector. The Dual-LUC assay was conducted according to previously described methods (Zhang et al. 2024). All primers used here were listed in Supplemental Table S1.

### Measurement of GA contents

The GAs contents of chilling- and TRV2-*PsGA2ox2* buds and transgenic Arabidopsis were measured with using liquid chromatography with tandem mass spectrometry (LC–MS/MS) as described previously with minor modifications (Chen et al. 2012). In briefly, about 2 g of samples were ground in liquid nitrogen and extracted with 1 mL 80% methanol (v/v) for 12 h in the dark at 4℃. Following centrifugation at 15,000 g for 10 min, GAs were analyzed by LC–MS/MS. [^2^H_2_] GA_1_ (1.0 ng·g^−1^), [^2^H_2_] GA_4_ (1.0 ng·g^−1^), [^2^H_2_] GA_8_ (1.0 ng·g^−1^), [^2^H_2_] GA_34_ (1.0 ng·g^−1^), [^2^H_2_] GA_12_ (1.0 ng·g^−1^), and [^2^H_2_] GA_53_ (1.0 ng·g^−1^) were used as internal standards.

### Statistical analysis

Statistical analysis was performed using GraphPad Prism version 7.0. The data were tested with Student’s *t*-test or one-way analysis of variance (ANOVA) with Tukey’s multiple comparisons test.

## Supplementary Information

Additional file 1: Fig. S1. Sequences alignment of PsGA2ox2, PsGA2ox8, and GA2ox homologs of other plants. AtGA2ox1 (AT1G78440), AtGA2ox2 (AT1G30040), AtGA2ox3 (AT2G34555), AtGA2ox4 (AT1G47990), AtGA2ox6 (AT1G02400), AtGA2ox7 (AT1G50960), AtGA2ox8 (AT4G21200), CsGA2ox-2 (A0A2H4X2V4), VvGA2ox2 (D7TK65), and VvGA2ox8 (A0A438EEH6). Underline indicated the conserved 2OG-FeII_Oxy domain HMM matrix of GA2ox, and navy blue showed the conserved base.

Additional file 2: Fig. S2. Tissue-specific expression of PsGA2ox2 in tree peony using qPCR. Tree peony plants after 21 DAC were transferred to greenhouse for 45 d, and the tissues were collected. The buds were a mix of 0, 7, 14 and 21 DAC. Data were shown as mean ± SD from three biological replicates (five buds in each replicate). Asterisk indicated the significant differences (Student’s *t*-test, **P < 0.01).

Additional file 3: Fig. S3. The relative expression levels of PsGA2ox8.1 after GA3 feeding using qPCR. Tree peony plants after 7 DAC were transferred to greenhouse, and the buds were treated with 200 mg·L^−1^ GA_3_, and then the buds were collected. Error bars indicated SE (*n* = 3). Asterisk indicated the significant differences (Student’s *t*-test, *P < 0.05, **P < 0.01).

Additional file 4: Fig. S4. Schematic diagram of PsGA2ox2 promoter sequence and truncated fragments based on the MYB-binding motifs by PlantCARE. The MYB-binding sites were marked using triangles.

Additional file 5: Fig. S5. Interaction between PsMYB65 and the promoter of PsGA2ox2 by Y1H. The promoter was truncated to four fragments, and inserted into pHIS2.1, respectively. Positive colonies were cultured on the SD/-Trp/-Leu/-His medium with 3-AT.

Additional file 6: Fig. S6. Dual-LUC assay to assess the regulatory effect of PsMYB65 on PsGA2ox2 expression, with the relative LUC/REN activities (B, C). (A) Schematic of reporter and effect vectors using dual-LUC assay. Ns indicated no significant difference (one-way ANOVA, *P* < 0.05).

Additional file 7: Fig. S7. Target prediction between PsmiR159b* and PsGA2ox2 by psRNAtarget.

Additional file 8: Fig. S8. Dual-LUC assay to validate the direct regulation of PsGA2ox2 by PsmiR159b.

Additional file 9: Fig. S9. The phylogenetic tree of ABA-responsive transcription factors including PsABF2a, PsABF2b (A), and PsABI5 (B), and the other homologs from the known plants constructed with MEGA 11.0 using the neighbor-joining method. Bootstrap values were 1000 replicates. Dots and triangle referred to the corresponding proteins of tree peony, and the accession numbers were in brackets.

Additional file 10: Fig. S10. Relative expression levels of ABA pathway-related genes including PsABF2a, PsABF2b, and PsABI5 after chilling treatments using qPCR. Error bars indicated SE (*n* = 3). Asterisks indicated statistically significant differences (Student’s *t*-test, *P < 0.05).

Additional file 11: Fig. S11. Endogenous GA1, GA4, GA8, and GA34 levels in PsMIR159b-silenced (STTM159b) and OE-MIR159b buds. Error bars indicate SD (*n* = 3). Asterisks indicated statistically significant differences (Student’s *t*-test, *P < 0.05). OE, overexpression; STTM159b, silencing of PsmiR159b.

Additional file 12: Table S1. The primer information used in this study.

## Data Availability

Data will be available from the corresponding author upon reasonable request.

## Abbreviations

*3-AT*: 3-Amino-1,2,4-triazole
*ABA*: Abscisic acid
*DAC*: Days after chilling
*DAI*: Days after infection
*EDR*: Endodormancy release
*EMP*: Embden-Meyerhof-Parnas
*GA*: Gibberellin acid
*LC–MS/MS*: Liquid chromatography with tandem mass spectrometry
*OE*: Overexpression
*qPCR*: Quantitative Real-time PCR
*RLM 5’-RACE*: RNA ligase-mediated 5’- RACE
*VIGS*: Virus-induced gene silencing
*Y1H*: Yeast one-hybrid
