# Supplementary figures and images for: PsGA2ox2 is a novel target of miR159 involved in endodormancy regulation in tree peony (Paeonia suffruticosa)
Source: Mol Hortic. 2026 May 12;6:35. doi: 10.1186/s43897-025-00220-9 (PMC13162426; doi:10.1186/s43897-025-00220-9)

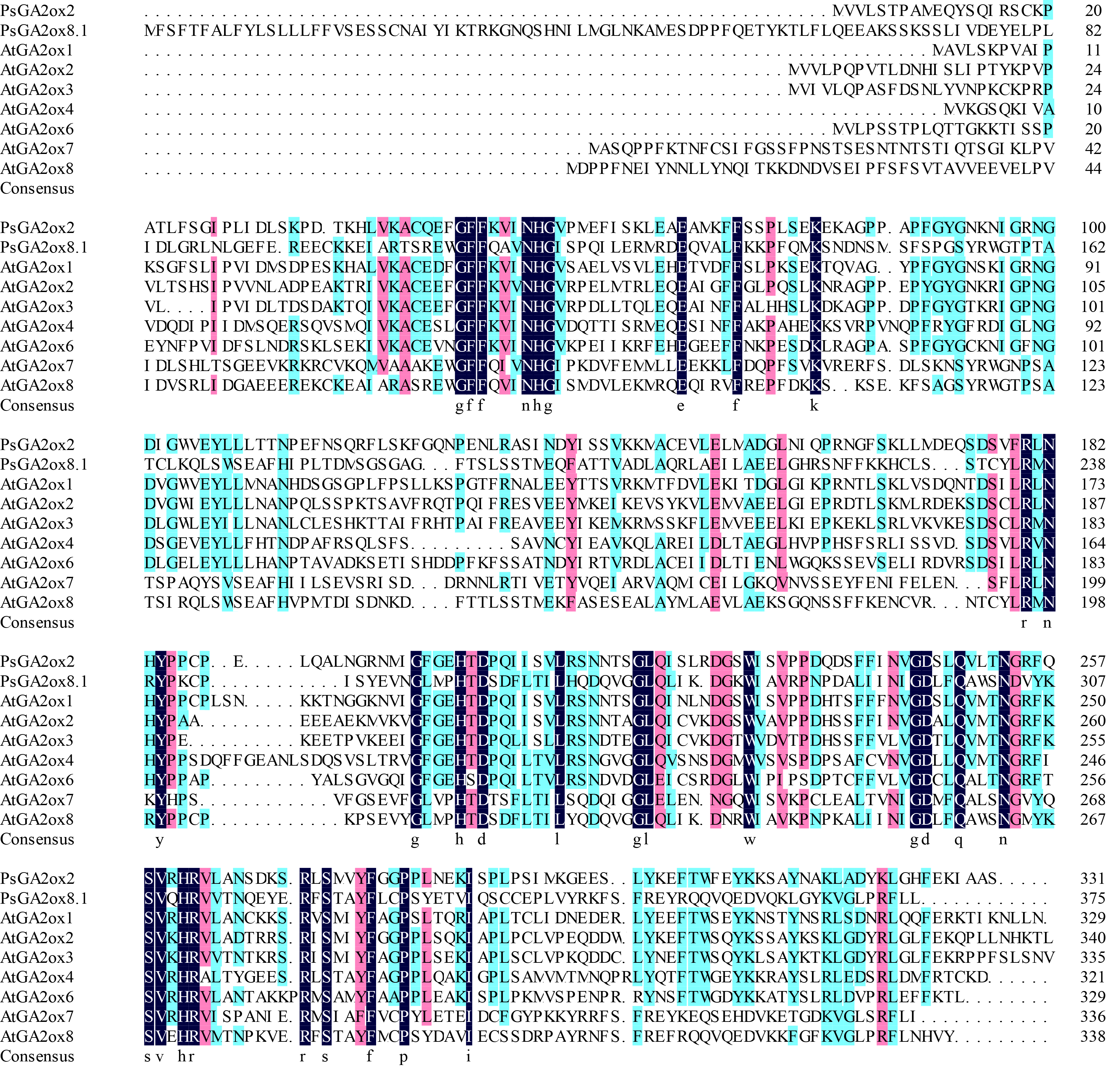

Supplement: Supplementary file 1 — Additional file 1: Fig. S1. Sequences alignment of PsGA2ox2, PsGA2ox8, and GA2ox homologs of other plants. AtGA2ox1 (AT1G78440), AtGA2ox2 (AT1G30040), AtGA2ox3 (AT2G34555), AtGA2ox4 (AT1G47990), AtGA2ox6 (AT1G02400), AtGA2ox7 (AT1G50960), AtGA2ox8 (AT4G21200), CsGA2ox-2 (A0A2H4X2V4), VvGA2ox2 (D7TK65), and VvGA2ox8 (A0A438EEH6). Underline indicated the conserved 2OG-FeII_Oxy domain HMM matrix of GA2ox, and navy blue showed the conserved base. [file 43897_2025_220_MOESM1_ESM.tif]

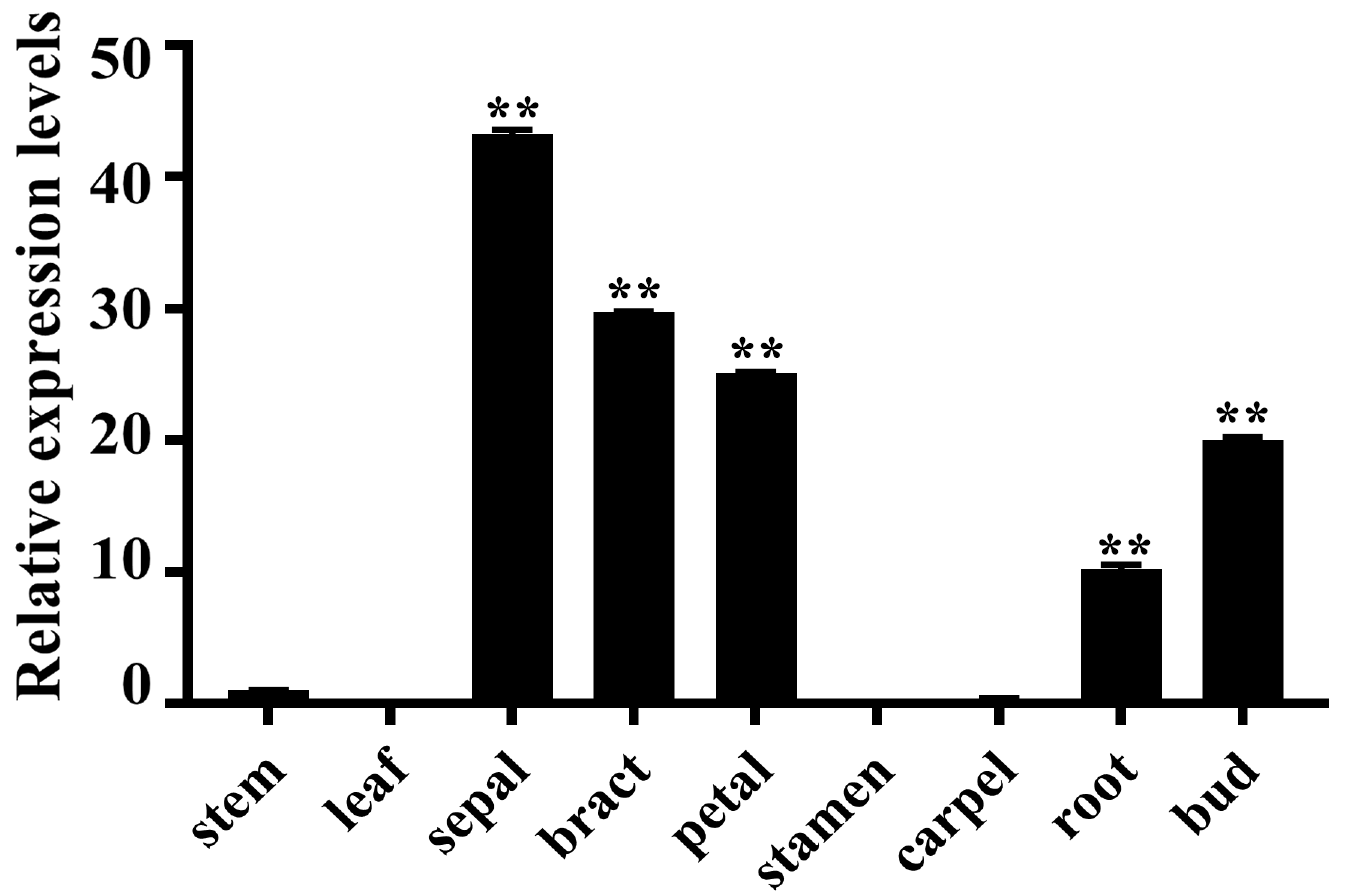

Supplement: Supplementary file 2 — Additional file 2: Fig. S2. Tissue-specific expression of PsGA2ox2 in tree peony using qPCR. Tree peony plants after 21 DAC were transferred to greenhouse for 45 d, and the tissues were collected. The buds were a mix of 0, 7, 14 and 21 DAC. Data were shown as mean ± SD from three biological replicates (five buds in each replicate). Asterisk indicated the significant differences (Student’s t-test, **P < 0.01). [file 43897_2025_220_MOESM2_ESM.tif]

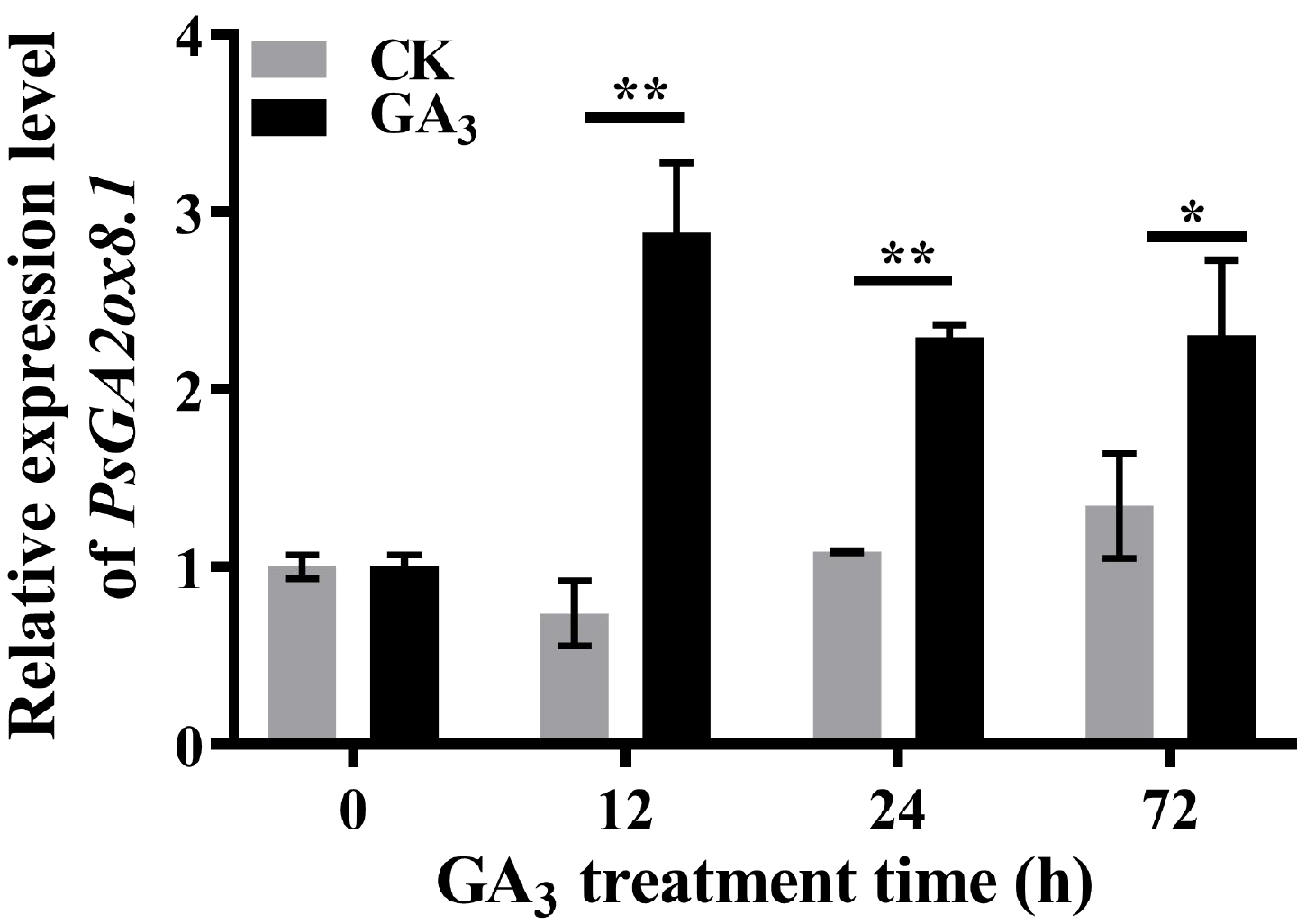

Supplement: Supplementary file 3 — Additional file 3: Fig. S3. The relative expression levels of PsGA2ox8.1 after GA3 feeding using qPCR. Tree peony plants after 7 DAC were transferred to greenhouse, and the buds were treated with 200 mg·L−1 GA3, and then the buds were collected. Error bars indicated SE (n = 3). Asterisk indicated the significant differences (Student’s t-test, *P < 0.05, **P < 0.01). [file 43897_2025_220_MOESM3_ESM.tif]

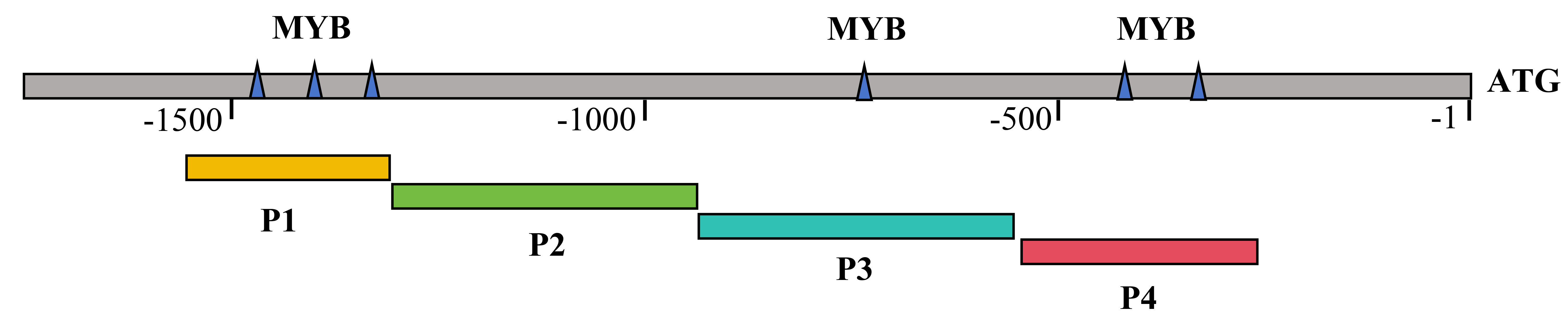

Supplement: Supplementary file 4 — Additional file 4: Fig. S4. Schematic diagram of PsGA2ox2 promoter sequence and truncated fragments based on the MYB-binding motifs by PlantCARE. The MYB-binding sites were marked using triangles. [file 43897_2025_220_MOESM4_ESM.tif]

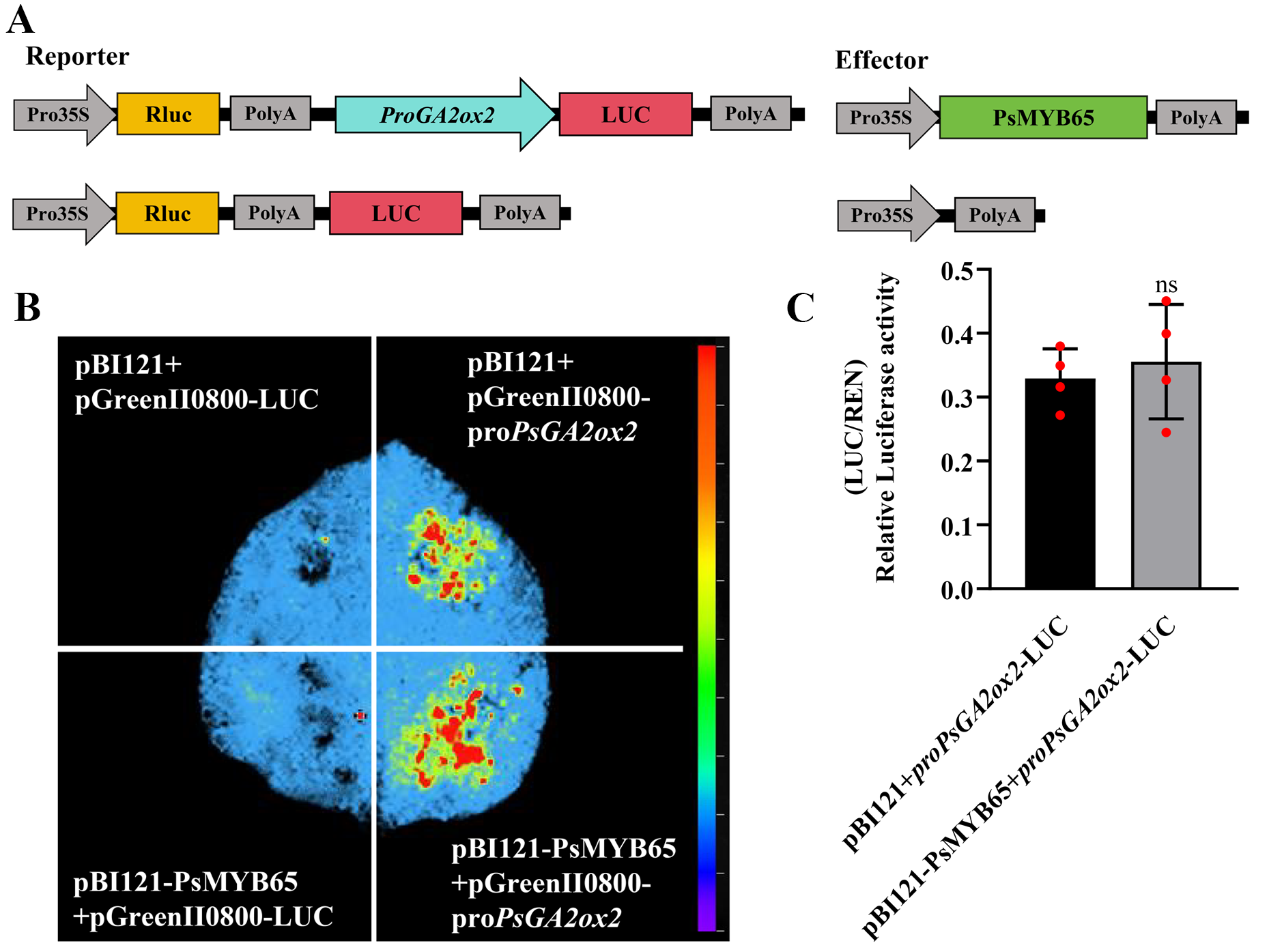

Supplement: Supplementary file 6 — Additional file 6: Fig. S6. Dual-LUC assay to assess the regulatory effect of PsMYB65 on PsGA2ox2 expression, with the relative LUC/REN activities (B, C). (A) Schematic of reporter and effect vectors using dual-LUC assay. Ns indicated no significant difference (one-way ANOVA, P < 0.05). [file 43897_2025_220_MOESM6_ESM.tif]

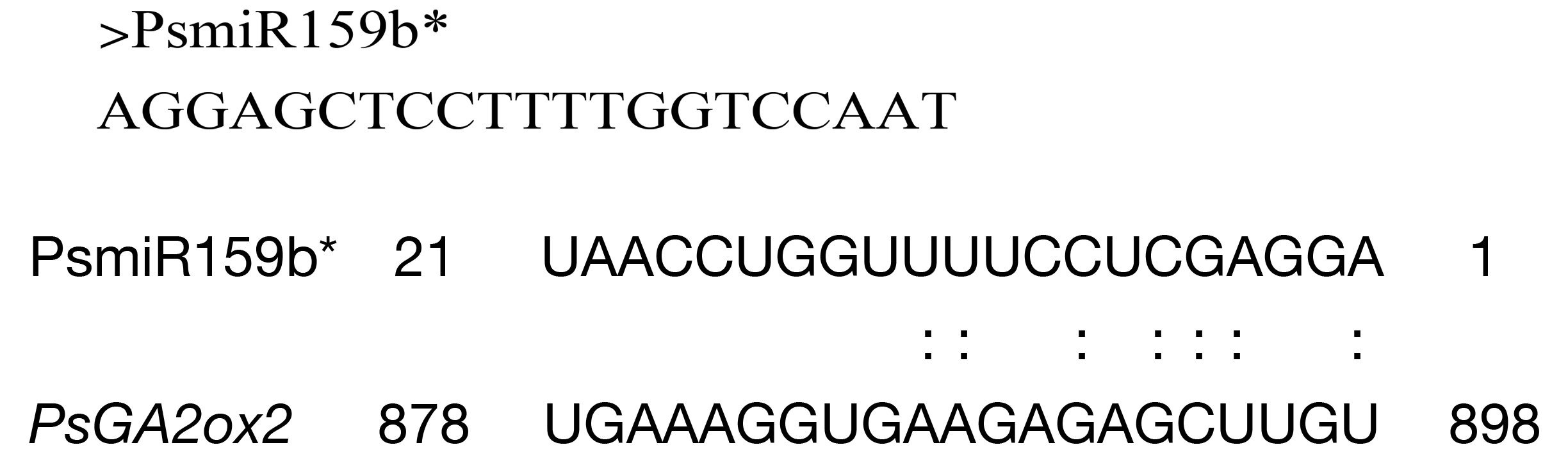

Supplement: Supplementary file 7 — Additional file 7: Fig. S7. Target prediction between PsmiR159b* and PsGA2ox2 by psRNAtarget. [file 43897_2025_220_MOESM7_ESM.tif]

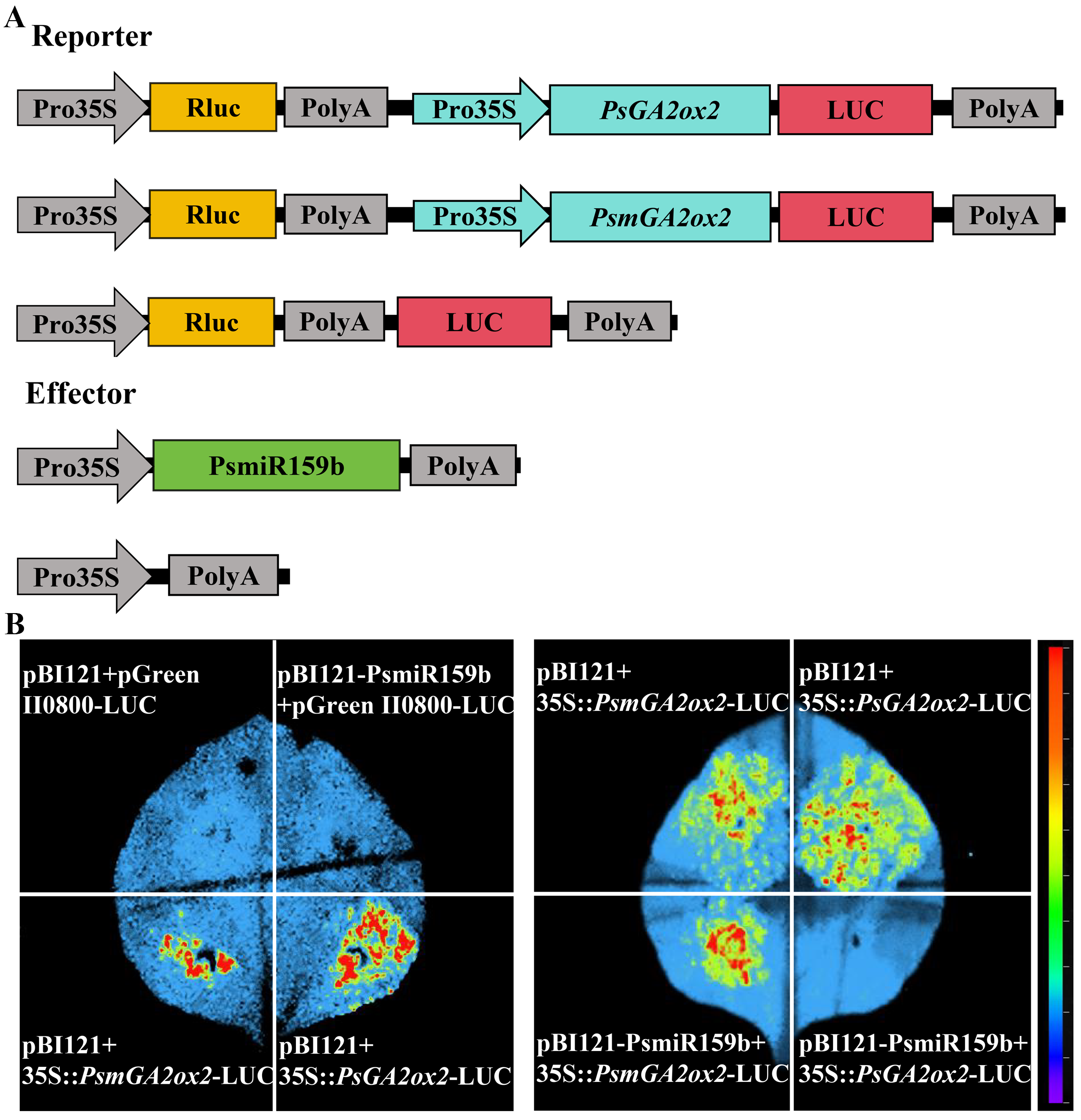

Supplement: Supplementary file 8 — Additional file 8: Fig. S8. Dual-LUC assay to validate the direct regulation of PsGA2ox2 by PsmiR159b. [file 43897_2025_220_MOESM8_ESM.tif]

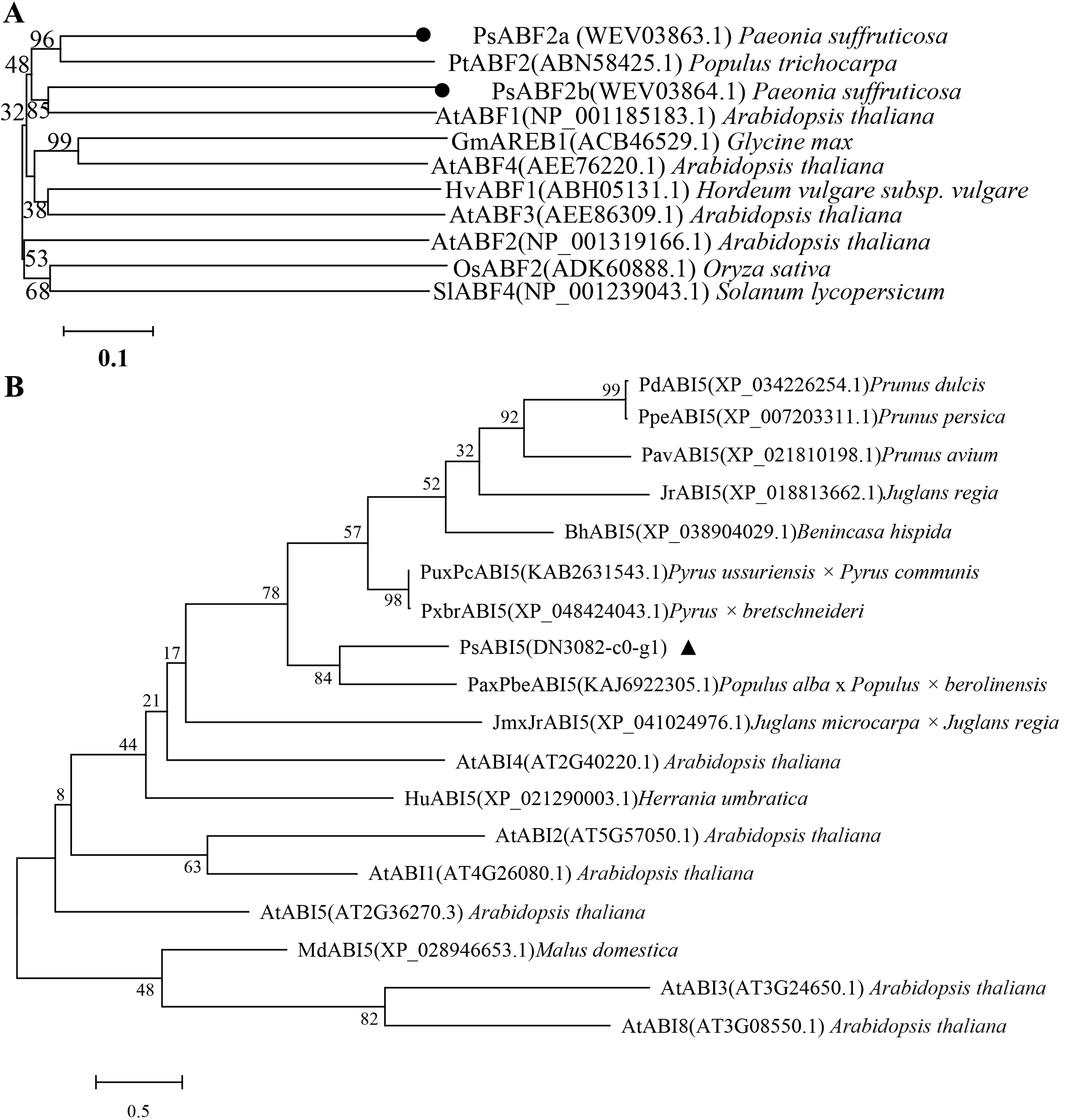

Supplement: Supplementary file 9 — Additional file 9: Fig. S9. The phylogenetic tree of ABA-responsive transcription factors including PsABF2a, PsABF2b (A), and PsABI5 (B), and the other homologs from the known plants constructed with MEGA 11.0 using the neighbor-joining method. Bootstrap values were 1000 replicates. Dots and triangle referred to the corresponding proteins of tree peony, and the accession numbers were in brackets. [file 43897_2025_220_MOESM9_ESM.tif]

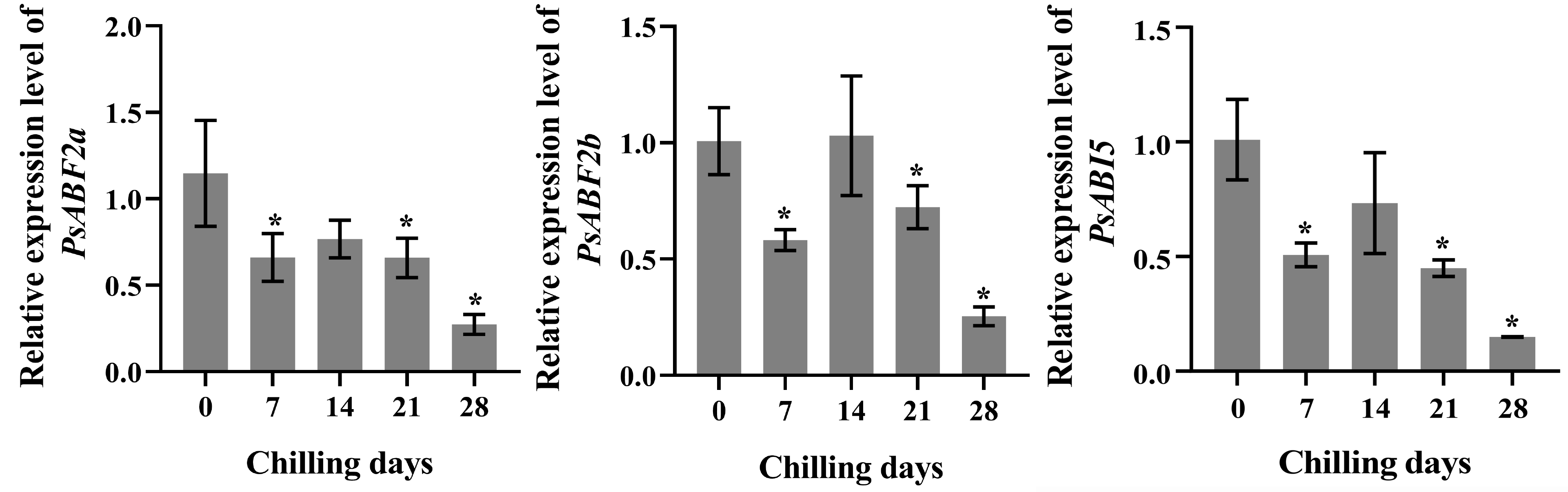

Supplement: Supplementary file 10 — Additional file 10: Fig. S10. Relative expression levels of ABA pathway-related genes including PsABF2a, PsABF2b, and PsABI5 after chilling treatments using qPCR. Error bars indicated SE (n = 3). Asterisks indicated statistically significant differences (Student’s t-test, *P < 0.05). [file 43897_2025_220_MOESM10_ESM.tif]

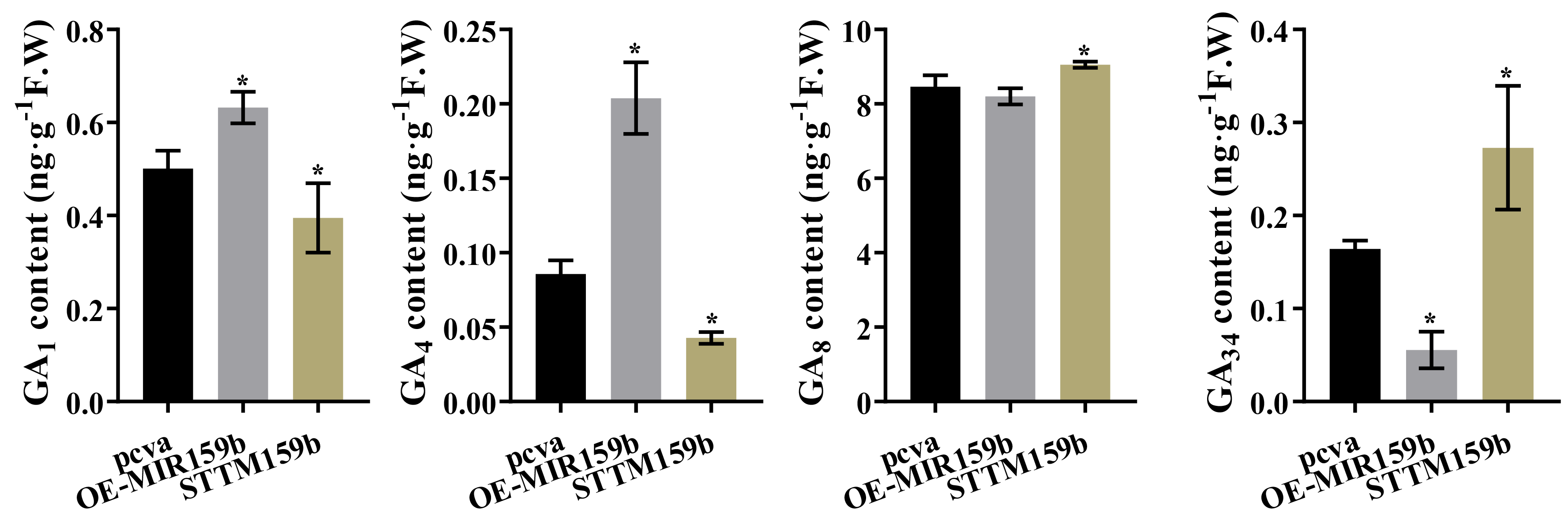

Supplement: Supplementary file 11 — Additional file 11: Fig. S11. Endogenous GA1, GA4, GA8, and GA34 levels in PsMIR159b-silenced (STTM159b) and OE-MIR159b buds. Error bars indicate SD (n = 3). Asterisks indicated statistically significant differences (Student’s t-test, *P < 0.05). OE, overexpression; STTM159b, silencing of PsmiR159b. [file 43897_2025_220_MOESM11_ESM.tif]
