# Supplementary material for: PsGA2ox2 is a novel target of miR159 involved in endodormancy regulation in tree peony (Paeonia suffruticosa)
Source: Mol Hortic. 2026 May 12;6:35. doi: 10.1186/s43897-025-00220-9 (PMC13162426; doi:10.1186/s43897-025-00220-9)
Supplement: Supplementary file 12 — Additional file 12: Table S1. The primer information used in this study. [file 43897_2025_220_MOESM12_ESM.docx]

**Additional file 12: Table S1. The primer information used in this study.**

|  |
| --- |

| Primer names | Sequences(5’-3’) | Purpose |
| --- | --- | --- |
| *PsGA2ox2*-qRT-F | CAATGGCGACATCGGGTGGG | qPCR |
| *PsGA2ox2*-qRT-R | AAGCCTGGACTTATCACTGTTTGC |  |
| *PsGA2ox8*-qRT-F | GAGTTACCGTTGGGGGACAC |  |
| *PsGA2ox8*-qRT-R | TGTGACCCAGTTCTTCTGCC |  |
| Ps-Actin-F | GTTCCAGCCATCACTAATCGG |  |
| Ps-Actin-R | GAATAGACCCTCCAATCCAGACAC |  |
| *PsmiR159b*-qRT-F | CGCGATTGGACTGAAGGGA |  |
| *PsmiR159b*-qRT-R | GTCGTATCCAGTGCAGGGTCCGAGGTATTCGCACTGGATACGACAGGAGC |  |
| *PsMYB65-*qRT-F | AACACCCGAATCAAGAGACG |  |
| *PsMYB65* -qRT-R | CCGAGTCCTTTCATCAGCAT |  |
| *PsABF2a*-qRT-F | GGGGCTGTGGAGAAGGTAGT |  |
| *PsABF2a*-qRT-R | CGTTTCTTCGCTCCTTGCTG |  |
| *PsABF2b*-qRT-F | GCAGAATGGGCATGGTAGGT |  |
| *PsABF2b*-qRT-R | TGCCGCTGACTCTCTGTTTT |  |
| *PsABI5-*qRT-F | TTGGACATGGGTGGTGTGAG |  |
| *PsABI5-*qRT-R | TTTCTTCTCAAGCTCGGCCA |  |
| *PsCYCD*-qRT-F | AGCAATGGGCACAGCAAAAG |  |
| *PsCYCD*-qRT-R | CACATCCACCAGAAACCCGA |  |
| *PsBG9-*qRT-F | TCATCTTCGCTCCGATGCTC |  |
| *PsBG9*-qRT-R | TCGGCCACAACTATGTCCAC |  |
| *PsRGL1-*qRT-F | ACCACGCCAAGATTTAGATG |  |
| *PsRGL1-*qRT-R | GTTGACTGAACTCGGTGAGG |  |
| *PsSVP-*qRT-F | TGATGGAAGAGAACAGGCGG |  |
| *PsSVP*-qRT-R | AACCCGAATATGGCAGTCCC |  |
| *PsmiR159b* | TGACAGAAGAGAGTGAGCACA | RT-PCR |
| *PsGA2ox -F* | CAATGGCGACATCGGGTGGG |  |
| *18S rRNA-F* | AGCCCAGACGACTATCACGC |  |
| *18S rRNA-R* | GCAAGAACACTCGCACCAGC |  |
| *PsGA2ox -R* | AAGCCTGGACTTATCACTGTTTGC |  |
| *GSP5’-GA2ox* | CAAGGAGTCACCAACATTAATGAAGAAGGAA | RLM 5’-RACE |
| *PsmiR159b-sense* | GAATTGTAATACGACACACTATAGTTTGGATTGAAGGGAGCTCTA | *In situ* hybridization |
| *PsmiR159b-antisense* | GAATTGTAATACGACACACTATAGATTGGATTGAAGGGAGCTCCT |  |
| *GA2ox-senseF* | GAATTGTAATACGACACACTATAGAACCCGACACTAAACACCTC |  |
| *GA2ox-senseR* | GCTTGCTGAACCCATTTC |  |
| *GA2ox-antiF* | AACCCGACACTAAACACCTC |  |
| *GA2ox-antiR* | GAATTGTAATACGACACACTATAGGCTTGCTGAACCCATTTC |  |
| TRV2-*PsGA2ox2*-F | aaggttaccgaattctctagaTGGTGGTTTTATCCACACCAGC | VIGS |
| TRV2-*PsGA2ox2*-R | gagacgcgtgagctcggtaccAACTTCACATGCCATCTTCTTGAC |  |
| pBI121-*PsGA2ox2*-F | gagaacacgggggactctagaATGGTGGTTTTATCCAAACCAGC | Arabidopsis transformation |
| pBI121-*PsGA2ox2*-R | ataagggactgaccacccgggTTATGAGGCTGCTATTTTCTCAAAGT |  |
| *PsmiR159b-GUS-F* | gagaacacgggggactctagaTTTGAGAGTGGAGCTCCTTGAAG | Transformation in *Nicotiana benthamiana* |
| *PsmiR159b-GUS-R* | ataagggactgaccacccgggCAAATGAATGAGGAGCTCCCTT |  |
| *PsGA2ox2-GUS-F* | gagaacacgggggactctagaATGGTGGTTTTATCCAAACCAGC |  |
| *PsGA2ox2-GUS-R* | ataagggactgaccacccgggTGAGGCTGCTATTTTCTCAAAGTG |  |
| *PsmGA2ox2-GUS-*F | gagaacacgggggactctagaTTTGCAAATATCTCTGAGAGATGGAAGCTGG |  |
| *PsmGA2ox2-GUS-*R | ataagggactgaccacccgggATTAATGAAGAAGGAATCCTGATCTGGTGGTAC |  |
| pGADT7-*PsMYB65*-F | gccatggaggccagtgaattcATGAGTCGATTAACTAATGATAGCGAC | Y187 |
| pGADT7-*PsMYB65*-R | cagctcgagctcgatggatccAGGTAGTTCAGACATTTGATACACAGTAGG |  |
| pHIS2.1-*proPsGA2ox*-P1-F | gactcactatagggcgaattcTGTTAATGTGAATTGGATCGTGC |  |
| pHIS2.1-*proPsGA2ox*-P1-R | ataatgccaggaattactagtTGTTTTGCCGTTTAGATAATTAATAGC |  |
| pHIS2.1-*proPsGA2ox*-P2-F | gactcactatagggcgaattcTAAATTAGAGAAAGGTATTCGATAATTTGC |  |
| pHIS2.1-*proPsGA2ox*-P2-R | ataatgccaggaattactagtAAAAATATTTTTAAAATAAAAAAAAAAATGAT |  |
| pHIS2.1-*proPsGA2ox*-P3-F | gactcactatagggcgaattcTTGGTTAACCTAGGATCTATCTCATTAAA |  |
| pHIS2.1-*proPsGA2ox*-P3-R | ataatgccaggaattactagtCCCATGACCTCTCTGTGGTCA |  |
| pHIS2.1-*proPsGA2ox*-P4-F | gactcactatagggcgaattcCTCATGGGCCATGGCCTG |  |
| pHIS2.1-*proPsGA2ox*-P4-R | ataatgccaggaattactagtTAGATTCCACTTTTAGTCTTCATTCATTAT |  |
| pHIS2.1-*proPsGA2ox*-F | gactcactatagggcgaattcCCCCAAGCTTGGATTGCG |  |
| pHIS2.1-*proPsGA2ox*-R | ataatgccaggaattactagtTGTTGCTGCTGAGTAAGAGGGTAA |  |
| pBI121-*PsMYB65*-F | gagaacacgggggactctagaATGAGTCGATTAACTAATGATAGCGAC | Dual-LUC |
| pBI121-*PsMYB65*-R | ataagggactgaccacccgggAGGTAGTTCAGACATTTGATACACAGTAGG |  |
| pBI121-*PsmiR159b*-F | gagaacacgggggactctagaTTTGAGAGTGGAGCTCCTTGAAG |  |
| pBI121-*PsmiR159b*-R | ataagggactgaccacccgggCAAATGAATGAGGAGCTCCCTT |  |
| pGreenII 0800-*proPsGA2ox*-F | gggccccccctcgaggtcgacCCCCAAGCTTGGATTGCG |  |
| pGreenII 0800-*proPsGA2ox*-R | agaactagtggatcccccgggTGTTGCTGCTGAGTAAGAGGGTAA |  |
| pGreenII 0800-35S::*PsGA2ox*-F | tttggagagaacacgcccgggATGGTGGTTTTATCCAAACCAGC |  |
| pGreenII 0800-35S::*PsGA2ox*-R | atctccaccgcggtggcggccgcTGAGGCTGCTATTTTCTCAAAGTG |  |
| pGreenII 0800-35S::*PsmGA2ox*-F | tttggagagaacacgcccgggATGGTGGTTTTATCCAAACCAGC |  |
| pGreenII 0800-35S::*PsmGA2ox*-R | atctccaccgcggtggcggccgcTGAGGCTGCTATTTTCTCAAAGTG |  |
